# Improvement in Mouse iPSC Induction by Rab32 Reveals the Importance of Lipid Metabolism during Reprogramming

**DOI:** 10.1038/srep16539

**Published:** 2015-11-12

**Authors:** Yangli Pei, Liang Yue, Wei Zhang, Yanliang Wang, Bingqiang Wen, Liang Zhong, Jinzhu Xiang, Junhong Li, Shaopeng Zhang, Hanning Wang, Haiyuan Mu, Qingqing Wei, Jianyong Han

**Affiliations:** 1 State Key Laboratories for Agrobiotechnology, College of Biological Sciences, China Agricultural University, Beijing, 100193, China

## Abstract

Induced pluripotent stem cells (iPSCs) have variable expression levels of a series of genes that affect their pluripotent potential, but the regulatory mechanisms controlling reprogramming remain unclear. By testing the efficiency of iPSC generation using Oct4, Sox2, Klf4 (termed OSK) plus one additional gene, we found that Rab32 improved reprogramming efficiency. We established a system for detecting the number and the size of lipid droplets and autophagosomes per cell for tracking their morphological changes during reprogramming. Our results showed that Rab32 increased lipid storage during the early and middle stages, and also increased autophagy during the middle stage of reprogramming. These findings were further confirmed by the up-regulation of lipid biosynthesis and autophagosome formation related genes, of which their expression could improve iPSC induction. The inhibition of lipid biosynthesis and autophagosome formation significantly reduced reprogramming efficiency, and the inhibition of lipid synthesis phenotype could be rescued by the overexpression of Rab32. In addition, the expression of pluripotency genes such as *Klf2, Nr5a2 and Tbx3*, was up-regulated by Rab32. These results demonstrated that Rab32 could improve the induction of iPSCs through the enhancement of lipid biosynthesis, highlighting the importance of lipid metabolism during reprogramming.

Induced pluripotent stem cells (iPSCs) can be obtained by the forced expression of four transcription factors, Oct4 (also known as Pou5f1), Sox2, Klf4, and c-Myc (termed OSKM) in somatic cells^1^,^2^. Some iPSC lines are capable of generating full-term mice via tetraploid blastocyst complementation, which is the most stringent test of pluripotency^3^,^4^,^5^. Human iPSCs also share a similar pluripotent metabolomic signature to embryonic stem cells (ESCs)^6^. However, the gene expression profiles of ESCs and iPSCs from mouse and human suggest that the gene expression signature of iPSCs is different from ESCs^7^, and different iPSC lines show different expression levels of pluripotency-associated genes from one another^8^,^9^. The quality of different iPSC lines is variable, and this determines their ability to undergo germ-line transmission and live birth following tetraploid complementation^9^,^10^. Although reprogramming efficiency can be enhanced by the inclusion of other reported factors in addition to pluripotency-related transcription factors, additional factors that play more specific roles in facilitating the reprogramming process remain to be uncovered^11^,^12^.

In this study, we comparatively analyzed the gene expression profiles of iPSCs with different quality. High-quality iPSCs can produce iPSC-derived mice by the tetraploid complementation and undergo germ line transmission, while low-quality iPSCs can only produce chimeras with low coat-color contribution. We selected high-quality iPSC lines such as the OSKT cell line (which was generated by the factors Oct4, Sox2, Klf4 and Tbx3) and the SKR cell line (Sox2, Klf4 and Nr5a2) and low-quality iPSC lines such as OSK (Oct4, Sox2 and Klf4) and SKRM (Sox2, Klf4, Nr5a2 and c-Myc), which have been reported in our previous studies^8^,^9^. To uncover factors that improve the reprogramming efficiency, candidate genes were selected based on their higher expression levels in high-quality iPSCs. We then assessed the efficiency of iPSC generation using OSK plus one additional candidate gene. We found that Rab32 was able to efficiently facilitate iPSC reprogramming, and additional studies were performed to explore the mechanism of this action. Our results indicated that Rab32 enhanced lipid synthesis and activated autophagy formation, which, in turn, regulated genes that also affected the efficiency of iPSC induction. The inhibition of lipid synthesis and autophagy formation by specific inhibitors could significantly reduce iPSC colony numbers, thus underscoring the importance of lipid metabolism during reprogramming.

## Results

### Rab32 Facilitates Mouse iPSC Induction

**Figure 1:**
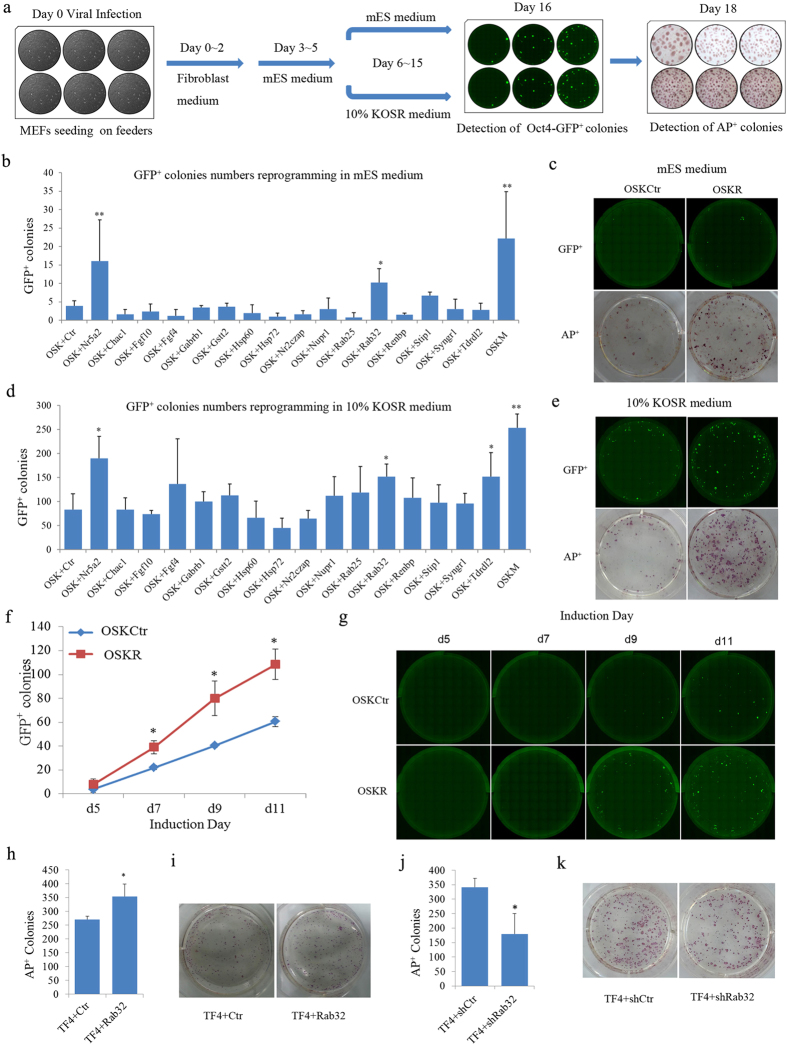
Rab32 Facilitates the Efficiency of Reprogramming. (**a**) Schematic representation of iPSC generation with different factor combinations. (**b**,**d**) The reprogramming efficiencies of factor combinations on the induction of iPSCs were quantified by determining the number of Oct4-GFP^+^ colonies in mES medium (**b**) and 10% KOSR medium (**d**) n = 4, the results of OSK+Ctr were used as control. *p < 0.05; **p < 0.01 by general linear model. (**c**,**e**) Full-well mosaic images of Oct4-GFP^+^ are shown for OSKCtr, OSKR and OSKN at day 16 and AP^+^ colonies at day 18 in mES medium (**c**) and 10% KOSR medium (**e**). (**f**) Characterization of Oct4-GFP^+^ dynamics for OSKCtr (blue plot) and OSKR (red plot) based on live imaging. (**g**) Representative of Oct4-GFP^+^ colonies during reprogramming (bar = 2 mm). (**h**,**i**) Reprogramming efficiency was increased by Rab32 in TF4 MEFs. (**j**,**k**) Rab32 knockdown reduces the number of AP^+^ colonies following the induction of iPSCs in TF4 MEFs. Data in (**f**,**h**,**j**) are represented as M ± SD (n = 3). The results of TF4 + Ctr, TF4 + shCtr and OSKCtr were used as controls respectively. *p < 0.05.

To better identify and characterize candidate genes that could facilitate the generation of iPSCs, we cloned a panel of full-length mouse genes into the pMXs retroviral vector^2^ and established a 6-well plate-based gene screening system (Fig. 1a) for reprogramming using various factor combinations. Mouse embryonic fibroblasts (MEFs) expressing a green fluorescent protein (GFP) reporter driven by an Oct4 promoter and enhancer (termed OG2 MEFs) were used in the screening. Reprogramming efficiency was evaluated by determining the number of Oct4 promoter driven GFP positive (Oct4-GFP^+^) and alkaline phosphatase-positive (AP^+^) colonies (for details see supplementary methods). The Oct4-GFP^+^ colonies generated from MEFs transfected with OSK and an empty retroviral vector was used as control (OSKCtr), and colonies generated by transfection with OSK and Nr5a2, a process which has been reported to enhance reprogramming efficiency^8^, were used as a positive control benchmark. To ensure the reliability of our results, we tested two culture conditions: standard mouse embryonic stem cell (mES) medium and KOSR medium, which has been reported to enhance iPSC induction^13^. Among the factors tested, we found that Rab32 markedly increased the number of Oct4-GFP^+^ colonies when it was co-infected with OSK into MEFs both in mES (Fig. 1b,c, p = 0.0384) and KOSR medium (Fig. 1d,e, p = 0.0205). Composite images of Oct4-GFP^+^ colonies at day 16 and representative images of AP^+^ colonies of OSKCtr and OSKR at day 18 in mES medium and KOSR medium are shown (Fig. 1c,e). In addition, the number of Oct4-GFP^+^ colonies based on live cell imaging (for details see supplementary methods) and the percentage of Oct4-GFP^+^ cells as determined using FACS analysis were used to measure the kinetics of reprogramming. We found that when *Rab32* was overexpressed, the number of Oct4-GFP^+^ colonies was increased (Fig. 1f,g) and that the percentage of Oct4-GFP^+^ cells was also significantly higher than the control groups (Supplementary Fig. 1a,b, p = 0.0175).

To further confirm the improvement of iPSC induction by Rab32, we used MEFs that carry doxycycline (Dox)-inducible OSKM reprogramming factors (termed TF4) for reprogramming^14^. The number of AP^+^ colonies was increased in the Rab32-overexpressing group compared to the control group when using KOSR medium containing Dox for 18 days (Fig. 1h,i, p = 0.0495). Meanwhile, the number of AP^+^ colonies was significantly reduced when *Rab32* was knocked down by a specific short hairpin RNA (shRNA) (Supplementary Fig. 1c) compared to control-treated TF4 cells (Fig. 1j,k, p = 0.04). Taken together, these results demonstrate that Rab32 was able to facilitate the induction of iPSCs.

### OSKR iPSCs are Pluripotent and Can Produce Chimeric Mice

**Figure 2:**
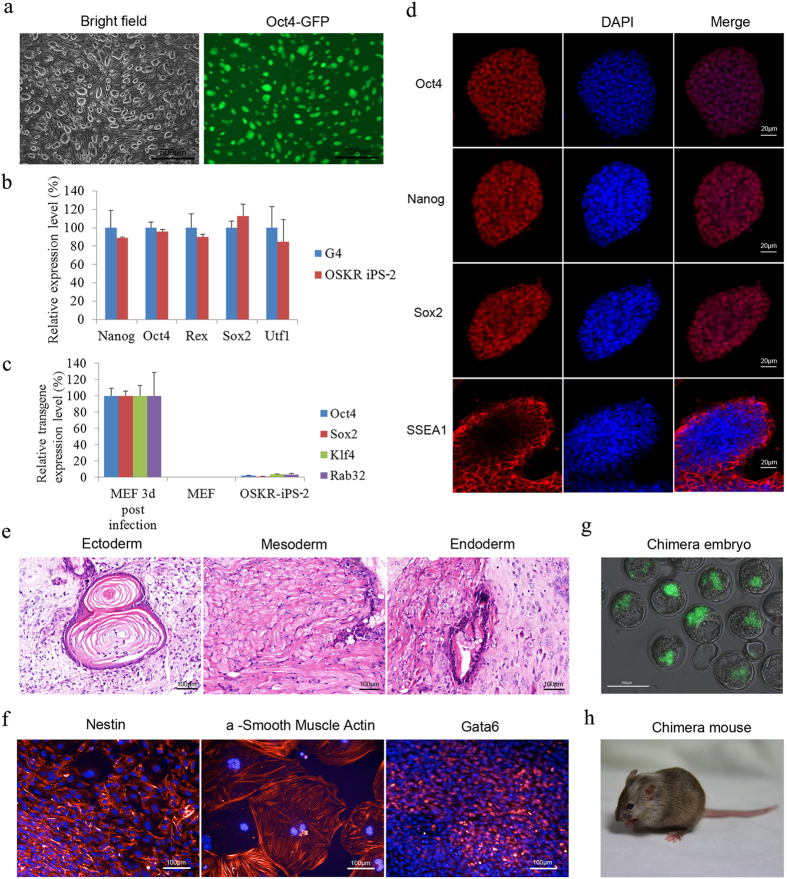
OSKR iPSCs Are Pluripotent and Can Produce Chimeric Mice. (**a**) Phase contrast and Oct4-GFP images of iPSC colonies generated from the retroviral transduction of OG2 MEFs with OSKR (bar = 500 μm). (**b**) q-PCR analysis of pluripotency genes expression in OSKR iPSC (OSKR iPS-2). The expression levels were normalized to those observed in G4 ESCs (n = 3). (**c**) Silencing of integrated transgenes in OSKR iPS-2. Three days after retroviral infection of MEFs with reprogramming factors, the expression of the exogenous factors could be detected using qPCR primers specific to the transgenes. In fully reprogrammed OSKR iPSCs, the expression of transgenes could not be detected (n = 3). (**d**) Immunofluorescence staining of pluripotency markers (Oct4, Nanog, Sox2 and SSEA1) in OSKR iPSCs (bar = 20 μm). (**e**) Hematoxylin and eosin staining of teratomas derived from OSKR iPSCs (bar = 100 μm). (**f**) EB-mediated *in vitro* differentiation assay performed on OSKR iPSCs. Differentiated cells stained positive for Gata6 (endoderm), Nestin (ectoderm), and α-smooth muscle actin (mesoderm) (bar = 100 μm). (**g**) Chimeric blastocysts after injecting OSKR iPSCs into 8 cell stage embryos (bar = 100 μm). (**h**) A two-week-old chimeric mouse was derived from OSKR iPSCs, and the photograph of the mouse was taken in our Laboratory Animal Center by Dr Yangli Pei. Relative expression was quantified using the comparative threshold cycle (Ct) method (2^−ΔΔCt^). The ΔCT was calculated using β-actin as internal control.

All OSKR iPSCs showed typical mESC-like morphologies and were Oct4-GFP^+^ (Fig. 2a). Quantitative real-time PCR (qPCR) analyses demonstrated that the expression levels of pluripotency marker genes, including *Nanog*, *Oct4*, *Rex1*, *Sox2*, *Utf1*, *Eomse*, *Esrrb*, *Klf2*, *Lin28a*, *Nr5a2*, *Sall4*, *Ssea1* and *Tbx3* in these cells were comparable to G4 ESCs (Fig. 2b and Supplementary Fig. 2a). Meanwhile, the expression of *Rab32* in OSKR iPSCs was comparable to ESCs (Supplementary Fig. 2b). The exogenous retroviral expression of the reprogramming factors were silenced (Fig. 2c), and OSKR iPSCs were AP-positive (Supplementary Fig. 2c). Our immunofluorescence staining of OSKR iPSCs further confirmed the protein levels of three master transcription factors (Oct4, Nanog and Sox2) and the mESC-specific surface marker SSEA-1 (Fig. 2d). The karyotypes of OSKR iPSCs were normal with 40 chromosomes (Supplementary Fig. 2d).

To further validate the pluripotency of OSKR iPSCs, we performed *in vivo* teratoma formation, *in vitro* differentiation and chimera production assays. The iPSCs were able to form teratomas *in vivo*, and hematoxylin and eosin (H&E) staining results confirmed the formation of three germ layers in the teratomas (Fig. 2e), and the iPSCs also formed embryoid bodies (EBs) *in vitro* (Supplementary Fig. 2e). The differentiated cells were positively stained for Nestin (ectoderm marker), Gata6 (endoderm), and a-smooth muscle actin (mesoderm) (Fig. 2f). Furthermore, we could derive chimeric embryos and mice from OSKR iPSCs (Fig. 2g,h), which was confirmed by PCR analysis (Supplementary Fig. 2f). These results indicated that OSKR iPSCs were pluripotent and fully competent to give rise to live animal. Next, we sought to explore the mechanism by which Rab32 improves the induction of iPSCs during reprogramming.

### The Overexpression of Rab32 Increases Lipid Storage and Activates Autophagy in MEFs

To systematically investigate the potential function of Rab32 during reprogramming and in stem cells, we examined its function in MEFs. We first hypothesized from other studies that Rab32 may participate in the regulation of apoptosis^15^, mitochondrial dynamics^16^, lysosome biogenesis^17^, melanosome maturation^18^,^19^,^20^, lipid storage^21^, and autophagosome formation^21^,^22^. Therefore, we focused on examining the role of these biological processes during reprogramming.

**Figure 3:**
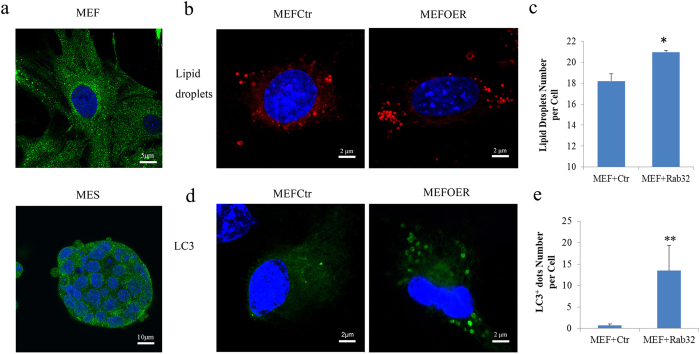
The Overexpression of Rab32 in MEFs Induces a Greater Number of Lipid Droplets and Autophagosomes. (**a**) Confocal immunofluorescence microscopy of MEFs double-labeled with polyclonal antibodies against Rab32 (green) (bar = 5 μm, up; bar  = 10 μm, down). (**b**) Nile red staining of lipid droplets in MEFs (bar = 2 μm). (**c**) Using Harmony software to calculate the number of lipid droplets, over-expression of *Rab32* results in a greater number of lipid droplets number compared to the control (n = 3, the result of MEFCtr was used as control, *p < 0.05). (**d**) Immunofluorescence staining of LC3^+^ dots in MEFs (bar = 2 μm). (**e**) The number of LC3^+^ dots per cell (n = 3, the result of MEFCtr was used as control, **p < 0.01) was quantified, and the overexpression of Rab32 induces more LC3 dots.

We first ensured that Rab32 was expressed in the MEFs and ESCs (Fig. 3a). MEFs were transfected with control vector (MEFCtr) or Rab32 (MEFOER), and we confirmed that Rab32 did not affect the proliferation of MEFs (details are provided in the methods and Supplementary Fig. 3). We used Nile red, Lyso-tracker, Mito tracker, and LC3 antibodies to stain for lipid droplets, lysosomes, mitochondria, and autophagosome, respectively. We then used an Operetta high content wide field fluorescence imaging system coupled to Harmony software to analyze the morphologies of cells, mitochondria, lipid droplets, lysosomes and autophagosomes (details are provided in the methods and Supplementary Fig. 4). The nuclear and cytoplasmic areas of the MEFs did not show marked changes resulting from increased levels of *Rab32* (Supplementary Fig. 5a). The mitochondrial structure were unperturbed based on our analysis using the Spot-Edge-Ridge (SER) texture features algorithm (Supplementary Fig. 5b and c). Lysosomes did not show significant changes (Supplementary Fig. 5d and e), while the number of lipid droplets per cells increased following *Rab32* overexpression (Fig. 3b,c, p = 0.0320). This increase did not affect the size of the lipid droplets (Supplementary Fig. 5f), and the average number of autophagosomes was also significantly increased following *Rab32* overexpression (Fig. 3d,e, p = 0.009); these results are similar to those from a previous study^22^. These data support the role of Rab32 in autophagy and lipid synthesis in MEFs during reprogramming.

### Rab32 Has Important Roles in Lipid Synthesis during Reprogramming

**Figure 4:**
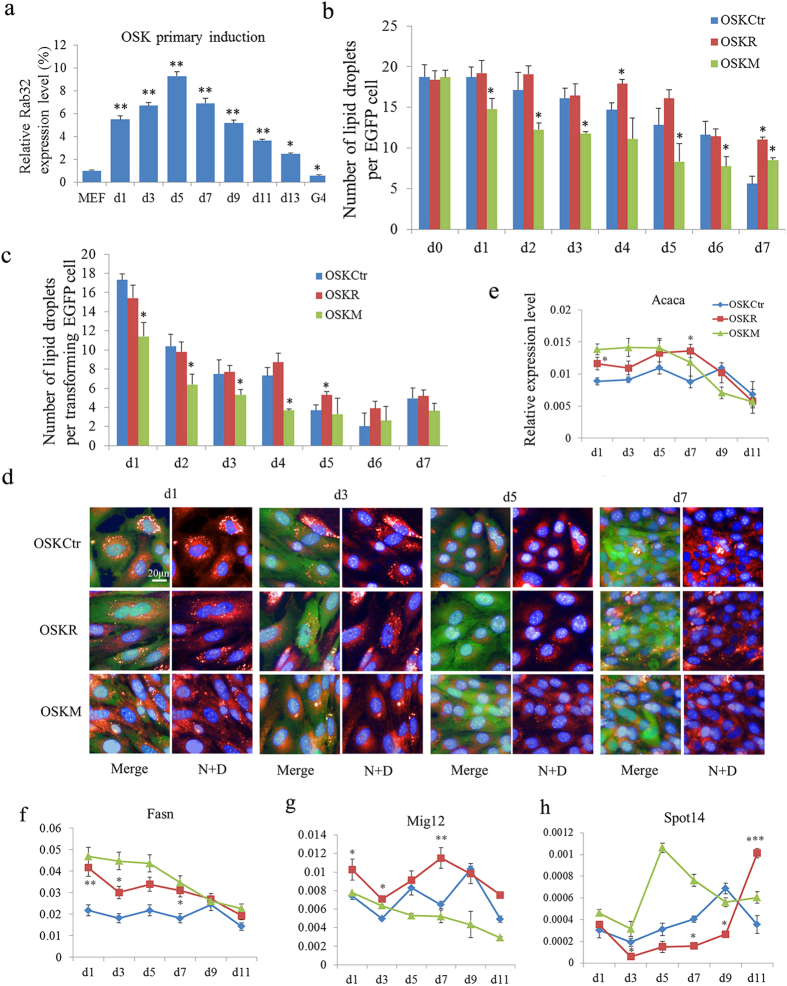
Rab32 Enhances Lipid Synthesis during Reprogramming. (**a**) Rab32 is progressively up-regulated in OSK-induced iPSCs until 5 days post virus infection compared to MEFs; Rab32 expression is then downregulated. Relative expression was quantified using the comparative threshold cycle (Ct) method (2^–ΔΔCt^). The ΔCT was calculated using β-actin, EF1-α and α-tubulin as internal control. *p < 0.05; **p < 0.01. (**b**) Lipid droplets dots per EGFP cell were calculated and compared to the MEFs induced with OSKCtr on the same day (n = 3, the results of OSKCtr were used as control, *p < 0.05). (**c**) Lipid droplets dots per transformed EGFP cell were calculated and compared with the MEFs induced with OSKCtr on the same day (n = 3, the results of OSKCtr were used as control, *p < 0.05). (**d**) Representative images showing the change in the number of lipid droplets during reprogramming. Lipid droplets were stained with Nile red, and the nuclei were stained with DAPI in EGFP cells seeded on feeders (bar = 20 μm,N: Nile red, D: DAPI).(**e**–**h**) The expression levels of *Acaca*, *Fasn*, *Mig12*, and *Spot14* were determined by qPCR on day 1, 3, 5, 7, 9 and 11 during reprogramming with OSKCtr, OSKR and OSKM. The data in Figure 4e–h, Fig. 5e–i, Fig. 6a–f are representative of three different experiments and are shown as M ± SD, n = 3, the results of OSKCtr were used as control, *p < 0.05, **p < 0.01. The gene expression profiles were expressed using the comparative CT (2^−ΔCT^) method. The ΔCT was calculated using β-actin, EF1-α, and α-tubulin as internal controls.

To understand the role of Rab32 in the induction of iPSCs, we examined the expression dynamics of Rab32 during reprogramming. Rab32 was up-regulated continuously until day 5, at which time it was then down-regulated but remained at a higher level than in MEFs reprogrammed with OSK (Fig. 4a). Because Rab32 regulated lipid storage and autophagy formation in MEFs, we examined the changes in lipid droplets and autophagosomes in OSKCtr, OSKR and OSKM iPSCs. To differentiate between feeder cells and reprogramming cells, we used MEFs that ubiquitously express enhanced GFP (EGFP) for iPSCs induction. During the course of reprogramming, the number of lipid droplets per EGFP-positive cell in all groups decreased gradually, but OSKR iPSCs showed a greater number of lipid droplets per cells compared to the OSKCtr and OSKM during the early and middle stages of reprogramming (Fig. 4b). However, the number of lipid droplets in transformed cells (whose karyoplasmic ratio (KR) more than 0.18; details are provided in Supplementary Fig. S6) showed a sharp decline one day after infection in all groups, and the cells began to accumulate lipids by day 7 (Fig. 4c,d). Furthermore, we analyzed the mRNA levels of various lipid synthesis-related genes during the process of iPSC formation. Interestingly, the expression levels of genes that control lipid synthesis such as *Fasn*, *Acaca*, *Mig12* and *Plin2* were higher in OSKR than OSKCtr during early (from day 1 to day 5) and middle stages (from day 5 to day 9) of iPSC formation. *Spot14*, a gene that can reduce the availability of malonyl-CoA^23^, was expressed at significantly lower levels during early and middle reprogramming stages in OSKR cells compared to OSKCtr and OSKM cells (Fig. 4e–h and Supplementary Table 1).

These results indicated that Rab32 up-regulated lipid synthesis-related genes to increase the number of lipid droplets per cells during early and middle stages of reprogramming. This suggests that the reprogramming process may require the consumption of large amounts of lipid, which can provide metabolites or energy. Previous studies have identified a critical function for autophagy during lipid metabolism^24^,^25^, and this led us to further investigate the effects of Rab32 on autophagy during reprogramming.

### Rab32 Increases Autophagosome Formation during Reprogramming

To differentiate between feeder cells and MEFs that were transduced with the reprogramming factors, we used RFP mitochondria-labeled MEFs for iPSC induction (details are descripted in the methods section). Autophagosomes appeared on the first day and reached their peak number on day 3 after induction in the OSKCtr, OSKR and OSKM groups (Supplementary Fig. 7a and b). Interestingly, the number and area of autophagosomes in OSKR cells were greater compared to the other two groups on days 5 and 7 (Supplementary Fig. 7a, b and c). Rab32 is required for the formation of autophagic vacuoles under basal conditions^22^. The above results suggested that *Rab32* might take part in the regulation of autophagy during early stages of reprogramming. During the middle stage of reprogramming, Rab32 might continue to drive autophagy and this was confirmed by the expression patterns of genes related to autophagosome formation. *Ulk1*, *Atg3*, *Atg4a*, *Atg4b*, *Map1lc3a* and *Map1lc3b* markedly increased their expression by day 5 or day 7 in OSKR cells (Supplementary Fig. 7d, e, f, g and Supplementary Table 2). When *Rab32* became silent during later stages of reprogramming, the number of autophagic vacuoles and genes expression returned to normal levels. mTOR, which can inhibit autophagy^26^, and *Tsc1*, *Tsc2*, which are critical negative regulators of mTORC1^27^, were expressed more highly in Rab32-expressing cells than in control cells (Supplementary Fig. 7h and i and Supplementary Table 3). The results indicated that Rab32 could enhance autophagosome formation during the middle stage of reprogramming. Previous reports have shown that the efficiency of iPSC induction cannot be significantly increased by rapamycin, an activator of autophagy, when added during middle stage of reprogramming^28^. This suggested that an increase in autophagy during the middle and late stages would not increase the efficiency of iPSC induction.

Although the above results indicated that Rab32 improves iPSC induction and increase lipid biosynthesis and autophagy during reprogramming, we wondered if lipid biosynthesis and autophagy are required for iPSC induction, and whether they enhance reprogramming.

### Lipid Biosynthesis and Autophagy Are Required for iPSC Induction

**Figure 5:**
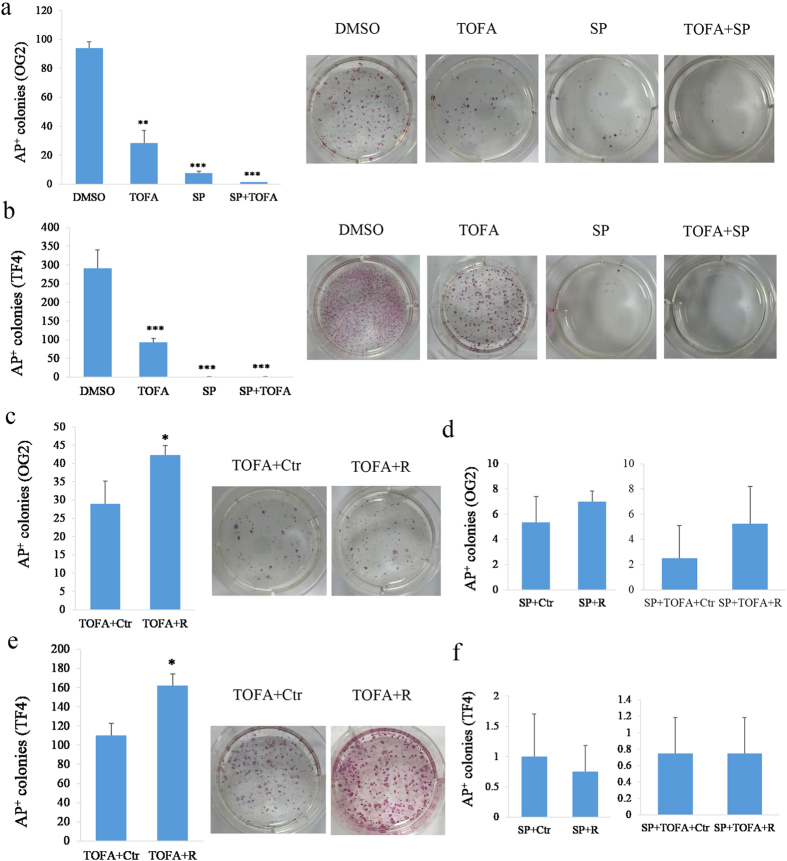
Rab32 Rescues the Reduction in iPSC Generation Produced by Inhibiting Lipid Synthesis during Reprogramming. (**a**) Inhibitors of autophagy and lipid synthesis reduce the efficiency of reprogramming with OSK in the OG2 MEFs. The results of DMSO treatment were used as controls, and the right side was the representative of AP^+^ colonies (n = 3, *p < 0.05, **p < 0.01,***p < 0.001). (**b**) Inhibitors of autophagy and lipid synthesis reduce the efficiency of reprogramming in TF4 MEFs. The results of DMSO were used as controls, and the right side was the representative of AP^+^ colonies (n = 3, *p < 0.05, **p < 0.01, ***p < 0.001). **(c**) Rab32 rescues the impact of reducing the programming efficiency caused by TOFA in the OG2 MEFs. The results of TOFA+Ctr were used as controls, and the right side was the representative of AP^+^ colonies (n = 3, *p < 0.05). (**d**) Rab32 cannot rescue the impact of reducing the programming efficiency caused by SP and SP+TOFA in the OG2 MEFs. The results of SP+Ctr and SP+TOFA+Ctr were used as controls (n = 3). (**e**) Rab32 rescues the reduction in programming efficiency caused by TOFA in TF4 MEFs. The results of TOFA+Ctr were used as controls, and the right side is representative of AP^+^ colonies (n = 3, *p < 0.05). (**f**) Rab32 cannot rescue the impact of reducing the programming efficiency caused by SP and SP+TOFA in the TF4 MEFs. The results of SP+Ctr and SP+TOFA+Ctr were used as controls (n = 3).

To determine the role of lipid biosynthesis and autophagy, we performed experiments using inhibitors specific to either of the processes during reprogramming to test their effects on iPSC induction. Autophagy can be blocked by the JNK inhibitor (SP600125)^29^. TOFA (5-(Tetradecyloxy)-2-furoic acid), which can block the synthesis of malonyl-CoA by acetyl-CoA carboxylase (ACC), is an inhibitor of fatty acid synthesis^30^. We used the two inhibitors on both OG2 and TF4 MEF cells to assess the impact on reprograming. We found that the reprogramming efficiency of both OG2 and TF4 MEFs into iPSCs were blocked following the inhibition of either lipid synthesis or autophagocytosis. Notably, the number of AP^+^ colonies was almost undetectable when autophagy and lipid synthesis were inhibited together (Fig. 5a,b). We next overexpressed Rab32 in cells treated with either inhibitor, and the results in both OG2 and TF4 cells showed that Rab32 could rescue the reduction of iPSC formation caused by the inhibition of lipid synthesis (Fig. 5c, p = 0.0191 and Fig. 5d, p = 0.006); but not by the reduction in autophagocytosis (Fig. 5e and f) during reprogramming. Furthermore, we tested the effects of overexpressing lipid synthesis and autophagy-related genes on reprogramming efficiency, and we found that Mig12 and Ulk1 facilitated the induction of iPSCs (Supplementary Fig. 8a). Silencing Spot14, a negative regulator for lipid biosynthesis, also resulted in an increase in reprogramming efficiency (Supplementary Fig. 8b). These results indicated that autophagy and lipid synthesis are required during reprogramming and that Rab32 primarily regulates lipid metabolism.

### Rab32 Affects Expression of Pluripotency Genes during Reprogramming

**Figure 6:**
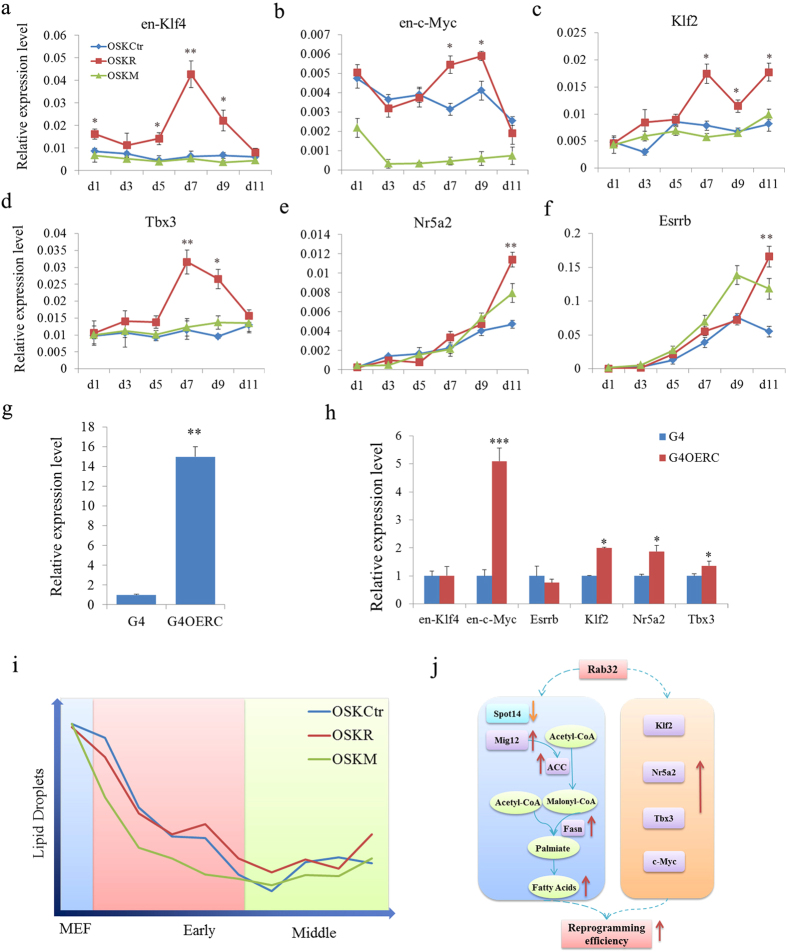
Rab32 Regulates Some Pluripotent Genes and the Proposed Mechanism of Rab32 during Reprogramming. (**a**–**f**) The expression levels of *en-Klf4*, *en-c-Myc*, *Klf2*, *Tbx3*, *Nr5a2* and *Esrrb* were determined by qPCR on day 1, 3, 5, 7, 9 and 11 during reprogramming with OSKCtr, OSKR and OSKM. The results of OSKCtr were used as control, *p < 0.05, **p < 0.01. (**g**) The expression level of *Rab32* in G4 cell transduced with two different vectors is increased compared to G4 cells, and G4OER cells express substantially higher levels compared to G4OERC cells. (**h**) The expression levels of *en-Klf4*, *en-c-Myc*, *Klf2*, *Tbx3*, *Nr5a2* and *Esrrb* were determined by qPCR. The results of G4 were used as control, *p < 0.05; **p < 0.01, ***p < 0.001. Relative expression (**g**,**h**) was quantified using the comparative threshold cycle (Ct) method (2^–ΔΔCt^). The ΔCT was calculated using β-actin, EF1-α and α-tubulin as internal controls. (**i**) Changes in lipid droplets during reprogramming. The number of lipid droplets per transformed EGFP cell declined sharply during early stages of reprogramming. OSKM cells metabolized most lipids, and Rab32 increased the storage of lipids in MEFs, but the lipids level was lower than in OSKCtr. Therefore, OSKR-reprogrammed cells metobolize more lipids than OSKCtr cells. A considerable amount of lipids must be metabolized during early stages of reprogramming, and transformed cells began to store lipids at middle stage. (**j**) After adding Rab32 with OSK, *Spot14* was down-expressed, while *Mig12, Acaca, Fasn* up-regulated, leading to a catalysis forming long-chain fatty acids during early and middle reprogramming stages. The additional expression of Rab32 also led to more expression of *c-Myc*, *Klf2*, *Tbx3* and *Nr5a2*. A combination of these results may be the mechanism of improvement in iPSC induction by Rab32.

To further identify whether Rab32 could influence the expression of pluripotency genes during reprogramming, we analyzed the expression of ESC pluripotency marker genes during reprogramming (Supplementary Table 4). Interestingly, endogenous *Klf4*, *c-Myc*, *Tbx3* and *Klf2* were expressed more highly in the MEFs transduced with OSKR compared to OSKCtr (Supplementary Table 4 and Fig. 6a–f). *Nr5a2*, *Sox2* and *Esrrb* showed slightly higher expression in OSKR compared to OSKCtr and OSKM cells during later reprograming stages. To further investigate whether Rab32 regulates these genes in ESCs, we constructed a Rab32 inducible overexpression vector, which was transfected into G4 ESCs termed G4OERC. q-PCR results confirmed that G4OERC overexpressed *Rab32* (Fig. 6g, p = 0.0090), and *c-Myc*, *Tbx3*, *Klf2* and *Nr5a2* were increased in G4OERC (Fig. 6h). These results suggested that Rab32 might regulate pluripotency factors during reprogramming.

## Discussion

Reprogramming efficiency can be enhanced by the inclusion of various factors in addition to those transcription factors responsible for pluripotency. These alternative factors either safeguard the pluripotency of ESCs or play more specific roles in facilitating the reprogramming process but do not maintain pluripotency after it is established^11^. In this study, we found that Rab32 was able to facilitate reprogramming efficiency by regulating lipid metabolism. These findings have implications for our understanding of the function of Rab32 during reprogramming.

Rab32 is a member of the Ras superfamily of small molecular weight G-proteins and has many functions in living cells^16^,^31^,^32^,^33^. By detecting a series of changes in MEFs, we found an increased number of phagosomes and lipid droplets following the overexpression of Rab32. Therefore we studied further the roles of lipid synthesis and autophagy during reprogramming. We did not find significant changes in apoptosis, mitochondrial dynamics, lysosomes or melanosomes following the overexpression of Rab32 in MEFs, which differed from some previous studies that examined the function of Rab32 in other contexts. These discrepancies are likely due to the function of Rab32 in different cell types and the treatment conditions^15^,^16^,^17^,^18^,^19^,^22^. For example, previous immunofluorescence analysis revealed that Rab32 overlap with the mitochondrial marker MitoTracker RedTM and is enriched in the P2 fraction in WI-38 (human lung fibroblast cell line) fibroblasts^16^. However, there have also been reports showing that Rab32 is predominantly localized to the endoplasmic reticulum (ER) in HeLa and COS cells^22^.

Somatic cell reprogramming to pluripotency is accompanied by a reset of metabolic processes, including lipid levels^34^. In this study, Rab32 participated in the regulation of autophagy and fatty acid metabolism during reprogramming and increased the number of lipid droplets and autophagosomes at early and middle stages. The expression levels of genes that control lipid synthesis and autophagy were also affected (Fig. 4 and supplementary Fig. 8). The number of lipid droplets per transformed EGFP cell declined sharply during early stages of reprogramming, and a greater number of lipid droplets were observed when Rab32 was used as an additional factor to reprogram MEFs into iPSCs (Fig. 6i). One property of the naïve mESCs state is lower number of lipid droplets^35^. So, the decreased number of lipid droplets per cells during reprogramming is not unexpected. The inhibition of both lipid synthesis and autophagy by specific inhibitors could reduce the induction of iPSCs; however, Rab32 could only rescue the effect of lipid metabolism inhibition. These results suggested that Rab32 mainly regulates lipid synthesis, which may be affected by autophagy^25^. This is supported by a previous study that showed the pharmacological inhibition of endogenous lipogenesis by the FASN inhibitor C57 to decrease reprogramming efficiency^36^.

The induction of iPSCs could also be improved by Mig12, which is an activator of ACC (acetyl-CoA carboxylase)^37^. ACC is a biotin-containing enzyme that catalyzes the carboxylation of acetyl-CoA to malonyl-CoA, which is the rate-limiting step in fatty acid synthesis. The silencing of endogenous Spot14, which can modulate lipogenesis by interacting with Mig12 to prevent its interaction with ACC^23^, also increased the number of AP^+^ colonies. These results demonstrated that lipid metabolism is required for reprogramming. After the addition of Rab32 to OSK, *Spot14* expression was down-regulated while *Mig12, Acaca* and *Fasn* were up-regulated leading to the formation of long-chain fatty acids. Thus, Rab32 might elevate lipid synthesis to provide additional lipids for cellular metabolism to elevate reprogramming efficiency (left side of Fig. 6j).

The expression levels of endogenous *Klf2*, *Nr5a2* and *c-Myc*. were up-regulated by Rab32 in MEFs during reprogramming and in ESCs. Increased expression of these factors can cooperate with other factors to more rapidly convert MEFs to iPSCs^2^,^8^,^9^,^38^,^39^ (right side of Fig. 6j). Metabolite fluxes also can be controlled by enzymes that are regulated by transcription factors including c-Myc^34^. However, the exact mechanism by which lipid metabolism facilitates reprogramming requires further investigation.

In summary, this study demonstrated that Rab32 could improve the induction of iPSCs by enhancing lipid synthesis, which highlights the importance of lipid metabolism during reprogramming.

## Methods

All experiments were performed in accordance with relevant guidelines and regulations. Mice were maintained in accordance with the Animal Experiment Standard of the Institute of Zoology, China Agricultural University. Briefly, mice were bred in a 12/12 h light/dark period and killed by cervical vertebral dislocation. All experiments were approved by the Animal Care and the Use Committees of the State Key Laboratories for Agrobiotechnology, College of Biological Sciences, China Agricultural University.

### Mouse Strains

B6D2-Tg (CAG/Su9-DsRed2, Acr3-EGFP) RBGS002Osb (no. 03743) mice, which express RFP mitochondria and GFP-labeled acrosomes, were used to provide MEFs with RFP-labeled mitochondria^40^. Oct4-GFP transgenic mice were used to provide OG2 MEFs. Transgenic mice that ubiquitously express enhanced GFP (Model Animal Research Center of Nanjing University) were used to provide EGFP MEFs.

### High-throughput Imaging

Cells cultured in 96-well plates were imaged from Day 1 to Day 11 with a PerkinElmer Operetta high-content wide-field fluorescence imaging system, coupled to Harmony software. Wells were imaged using a 20× objective lens, in a single focal plane across each plate. The bottom of each well was detected automatically by the Operetta focusing laser, and the focal plane calculated relative to this value. Hoechst emission (405 nm) was imaged for 50 ms; EGFP emission (488 nm) was imaged for 100 ms; Lyso-Tracker emission (542 nm) was imaged for 200 ms; and Nile red emission (542 nm) was imaged for 50 ms. A totla of 83 fields of view were imaged per well, with an identical pattern of fields used for every well. This pattern was designed to avoid imaging cells in the area targeted by the dispensers.

### Image Analysis

Modified Columbus (PerkinElmer) image analysis algorithms were used throughout. Nuclei were detected using a modified “find nuclei” algorithm as blue (Hoechst) fluorescent regions >30 μm^2^ with a split factor of 7.0, an individual threshold of 0.40, and a contrast >0.10 (method B). Cytoplasm were detected using a modified “find cytoplasm” algorithm as green (EGFP) fluorescent regions >100 μm^2^, with a split factor of 7.0, an individual threshold of 0.40, and a contrast >0.10 (method B). EGFP-negative objects were removed to ensure that only reprogrammed cells were analyzed. Border objects were excluded to ensure that only whole nuclei were analyzed. The phenotypic lyso-tracker spot and Nile red spot characteristics were detected using a modified “find spots” algorithm, as well as fluorescent spots with a relative spot intensity >0.090 and a splitting coefficient of 1.0 (method A). The area of the nuclei, cytoplasm and spot per spot, the number of spots per cell were exported as average value per well.

The transformed EGFP cells, whose karyoplasmic ratio (KR) was greater than 0.18, were analyzed next (KR = Nuclear area/cytoplasmic area; details are provided in Supplementary Fig. S6).

### Fast Gene Expression Analysis Using EvaGreen on the BioMark HD System

Intensity of fluorescence, loading uniformity, and the performance of reporter dyes in the micro-fluidic qPCR assays were treated using 96 × 96 dynamic arrays. Tests were performed using the universal mouse cDNA from OSKCtr, OSKR, OSKM on day 1, day 3, day 5, day 7, day 9, and day 11 after specific target amplification (STA). The procedures and operations were performed as detailed in the “Fluidigm Real-Time PCR Analysis Software User Guide”. Fluidigm Real-Time PCR Analysis Software v 3.0 was used to analyze the results.

Primer sequences for this section are listed in Supporting Information Table S5. We tested five reference genes: β-actin, EF1-α, α-tubulin, β-tubulin and B2m. Using geNorm analysis, we confirmed three normalization genes (β-actin, EF1-α, and α-tubulin) for further analysis.

### Inhibition of Lipid Synthesis and Autophagocytosis during Reprogramming

Autophagy can be blocked by the JNK inhibitor, SP600125^29^ and TOFA (5-(Tetradecyloxy)-2-furoic Acid) is an inhibitor of fatty acid synthesis that blocks the synthesis of malonyl-CoA by acetyl-CoA carboxylase (ACC)^30^. In this experiment, we used both OG2 and TF4 cells as original cells for reprograming. The OG2 MEFs were inducted with OSK, and TF4 cells were cultured with DOX. Simultaneously, they were both treated with inhibitors (control group: DMSO; SP group:10 μM SP600125; TOFA group: 10 μM TOFA; and SP + TOFA group: 10 μM SP600125 and 10 μM TOFA).

### Statistical Analysis

The results are presented as the mean ± standard deviation (MS ± D). Significant differences between groups in Oct4-GFP^+^ colonies number were determined using a general linear model with Statistical Analysis System (Version 8 of the SAS System). The significant differences between other results in this article were analyzed using Student’s t-tests.

## Additional Information

**How to cite this article**: Pei, Y. *et al*. Improvement in mouse iPSC Induction by Rab32 Reveals the Importance of Lipid Metabolism during Reprogramming. *Sci. Rep*. **5**, 16539; doi: 10.1038/srep16539 (2015).

## Supplementary Material

Supplementary Information
