# Supplementary material for: Improvement in Mouse iPSC Induction by Rab32 Reveals the Importance of Lipid Metabolism during Reprogramming
Source: Sci Rep. 2015 Nov 12;5:16539. doi: 10.1038/srep16539 (PMC4642270; doi:10.1038/srep16539)
Supplement: Supplementary Information [file srep16539-s1.doc]

**Improvement in Mouse iPSC Induction by Rab32 Reveals the Importance of Lipid Metabolism during Reprogramming**

Yangli Pei, Liang Yue, Wei Zhang, Yanliang Wang, Bingqiang Wen, Liang Zhong, Jinzhu Xiang, Junhong Li, Shaopeng Zhang, Hanning Wang, Haiyuan Mu, Qingqing Wei, Jianyong Han*

**Supplementary Information**

**Supplementary Data**


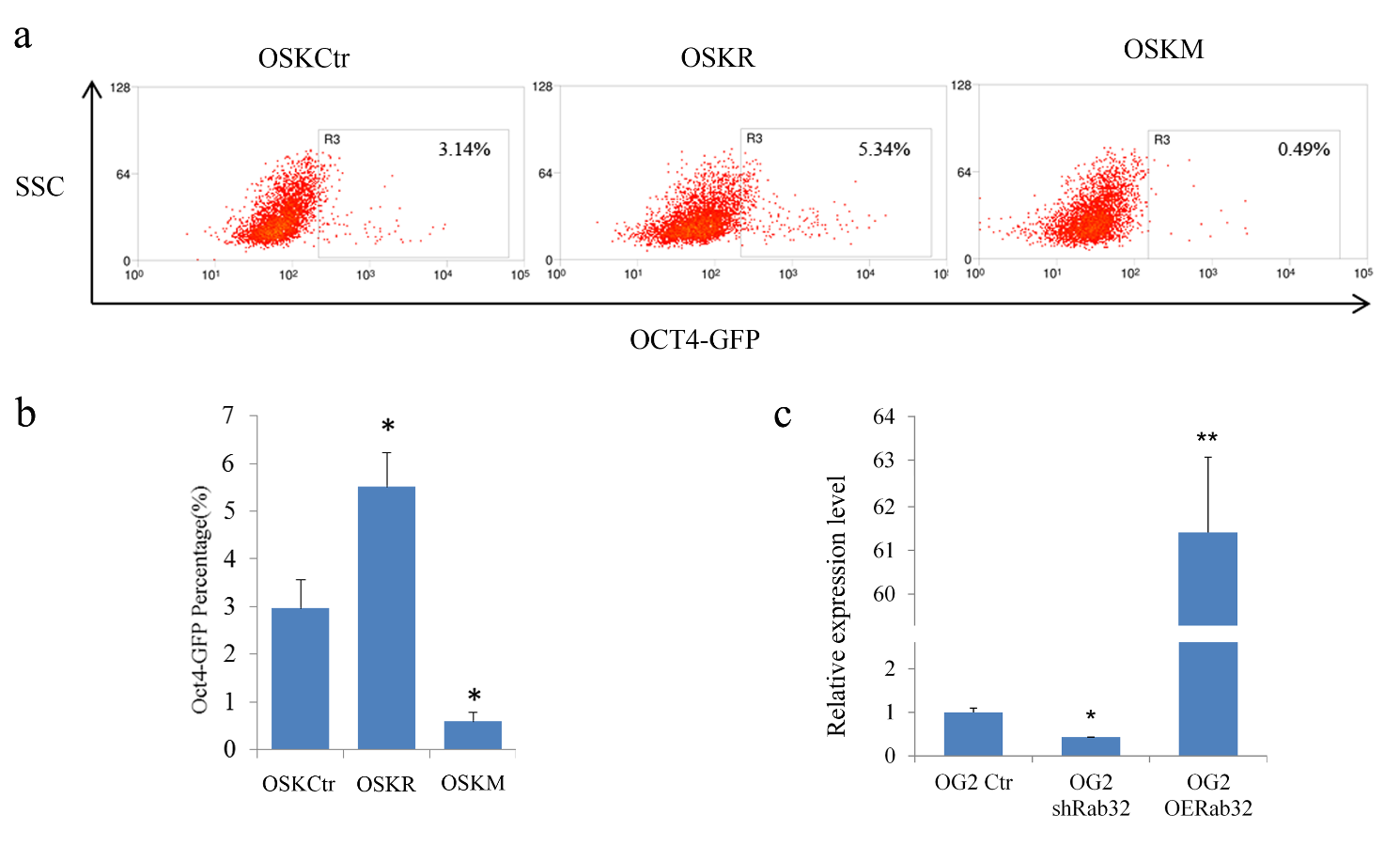


**Supplementary Figure 1. Rab32 facilitates the reprogramming efficiency.**

(**a, b**) Using FACS analysis to determine the percentage of Oct4-GFP positive cells on 7 days after MEFs being retroviral transduced with OSKCtr, OSKR and OSKM (n = 3, the results of OSKCtr were used as control. *p < 0.05). The representative FACS analysis image are shown in a.

(**c**) Using q-PCR to analyze the relative expression levels of Rab32 after shRab32 (short hairpin RNA specifically knock down Rab32) and pMXs-Rab32 vectors after being transduced into OG2 MEFs (n = 3, the results of OG2 Ctr were used as control. *p < 0.05, ** p < 0.01).


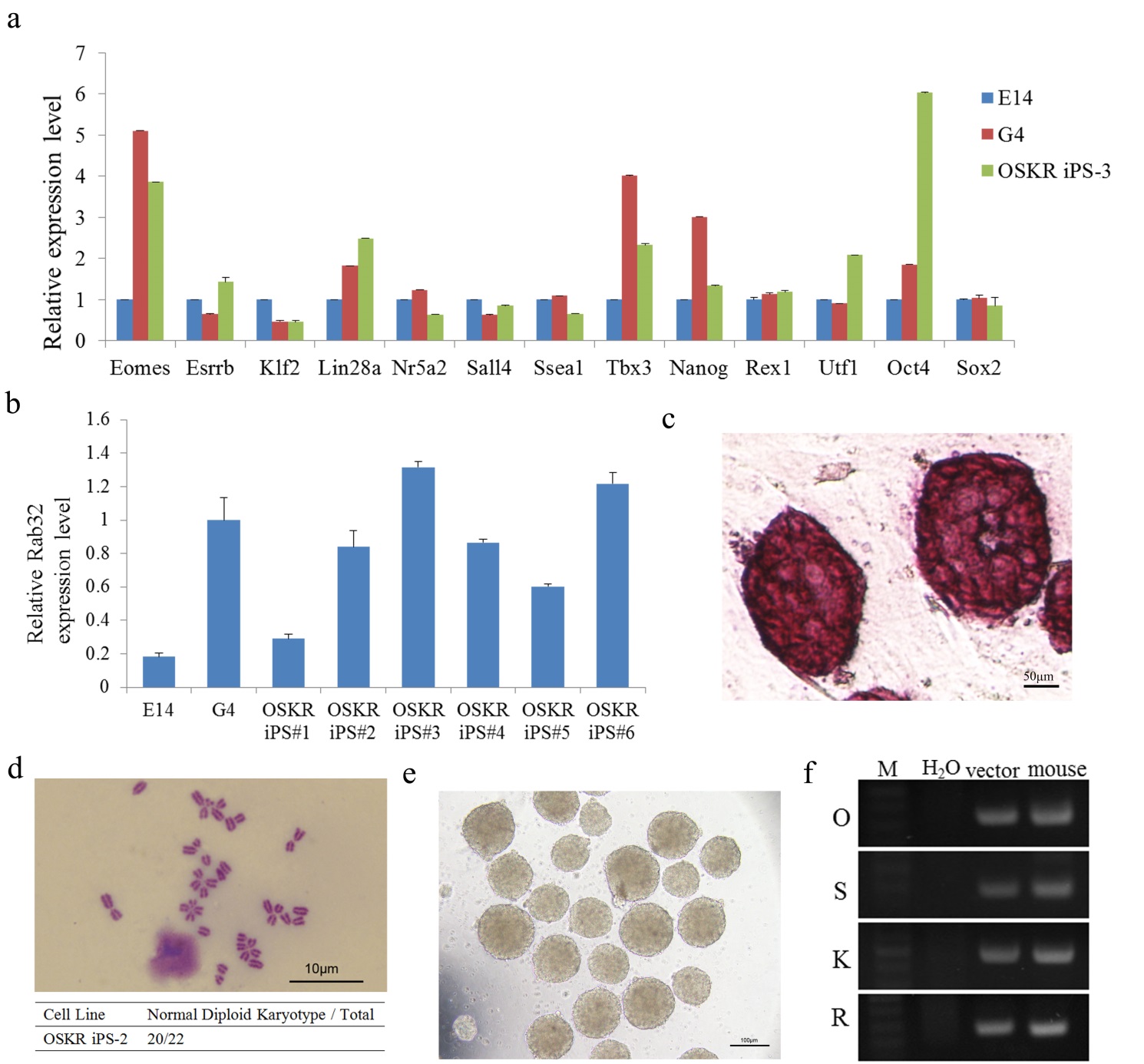


**Supplementary Figure 2. OSKR iPSCs are pluripotent.**

(**a**) q-PCR analysis of pluripotency genes expression in E14, G4, OSKR iPS-3. The expression levels were normalized to those observed in E14 ESCs (n = 3).

(**b**) The expression of Rab32 in OSKR iPSCs was normal detected by q-PCR (n = 3).

(**c**) OSKR iPSCs were AP positive (bar = 50 µm).

(**d**) The karyotypes of OSKR iPSCs were normal, with 40 chromosomes (bar = 10 µm), and the karyotyping analysis results of OSKR iPS-2 are shown below.

(**e**) OSKR iPSCs could form embryoid bodies (EBs) in vitro (bar = 100 µm).

(**f**) Genome of chimeric mice was detected with PCR primers specific to the transgenes, and the PCR results confirmed that the mice were from OSKR iPSCs. M (marker), O (Oct4), S (Sox2), K (Klf4), R (Rab32).

Relative expression was quantified using the comparative threshold cycle (Ct) method (2^–ΔΔCt^). The ΔCT was calculated using β-actin as an internal control.


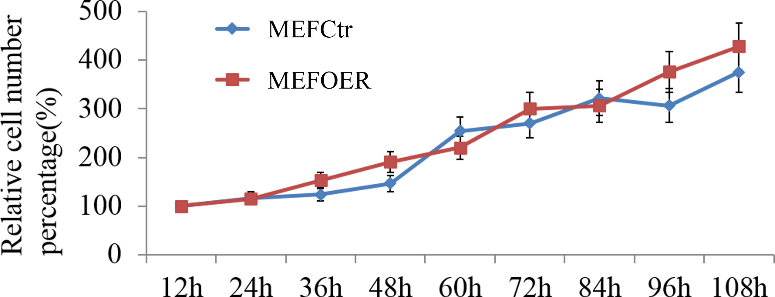


**Supplementary Figure 3. Extra-expression of Rab32 did not affect MEF proliferation.** MEFs were transfected with control vector (MEFCtr) or Rab32 (MEFOER), and then the cells were cultured in Biostation CT (Nikon) for 5 days following transfection. Cell images were automatically acquired every 12 hours by a BioStation CT, and the system’s software calculated the cell number and revealed that Rab32 did not affect the proliferation of MEFs.


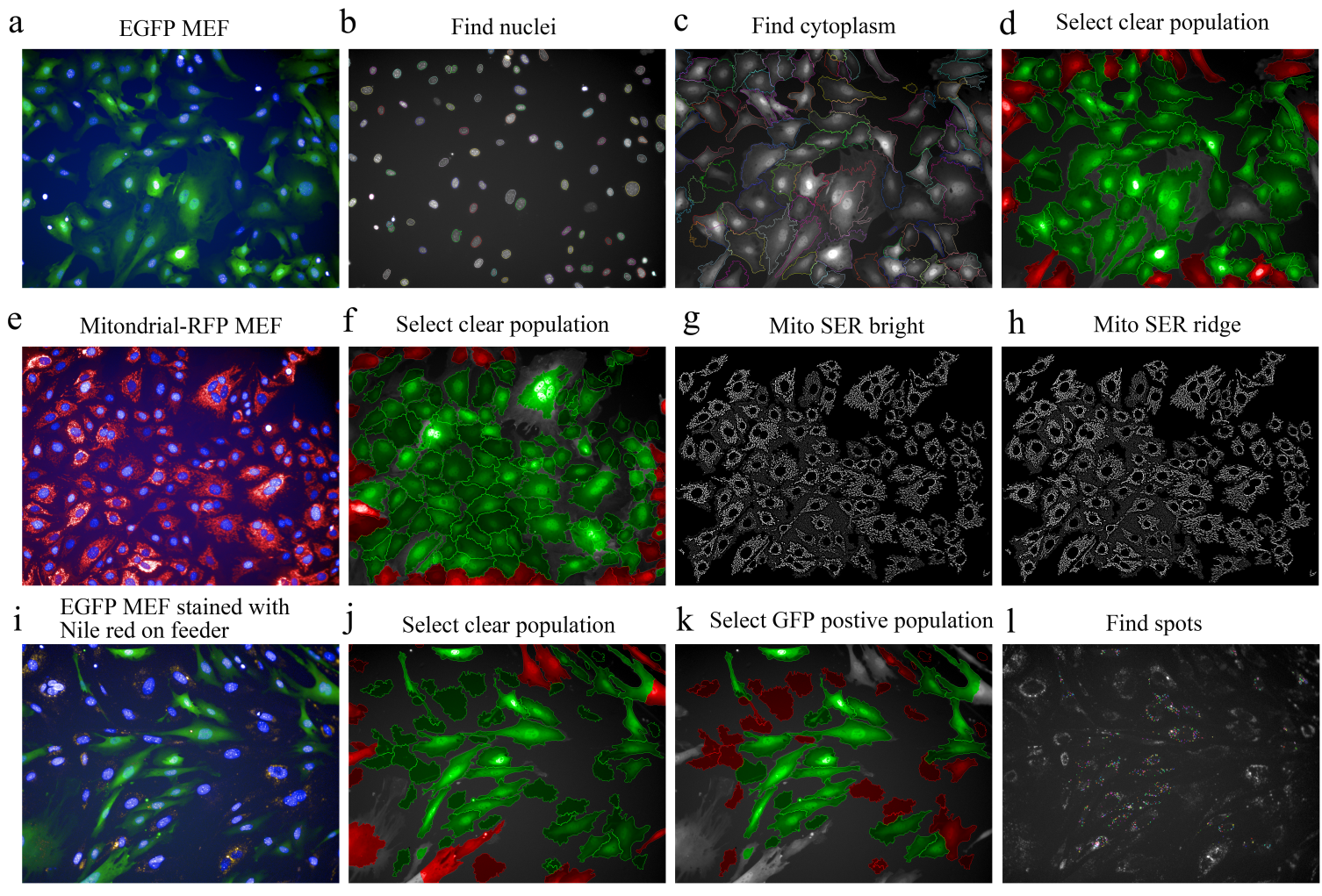


**Supplementary Figure 4. Examples of MEFs imaged by the Operetta system and analyzed by harmony software.**

(**a**) An example of EGFP cells imaged by the Operetta system, and the cells were analyzed by Harmony software (PerkinElmer) to identify the nucleus (**b**) and cytoplasm (**c**). The border objects were excluded to ensure only whole nuclei were analyzed (**d**).

(**e**) An example of mitochondrial-RFP cells imaged by the Operetta system. The border objects were excluded to ensure only whole nuclei were analyzed (**f**). Then the intensity structure of mitochondrial was analyzed with the Spot-Edge-Ridge (SER) texture features algorithm, including spot edge ridge texture analysis (**g**) and spot edge bright texture analysis (**h**).

(**i**) An example of EGFP cells seeded on no-fluorescence feeder cells imaged by the Operetta system and analyzed by Harmony software (PerkinElmer) to exclude border objects (**j**), (**k**) then GFP negative objects were removed to ensure that only EGFP cells were analyzed. (**l**) The phenotypic Nile red positive spots of cells after **j** and **k** selected characteristic were detected using a modified “find spots” algorithm.


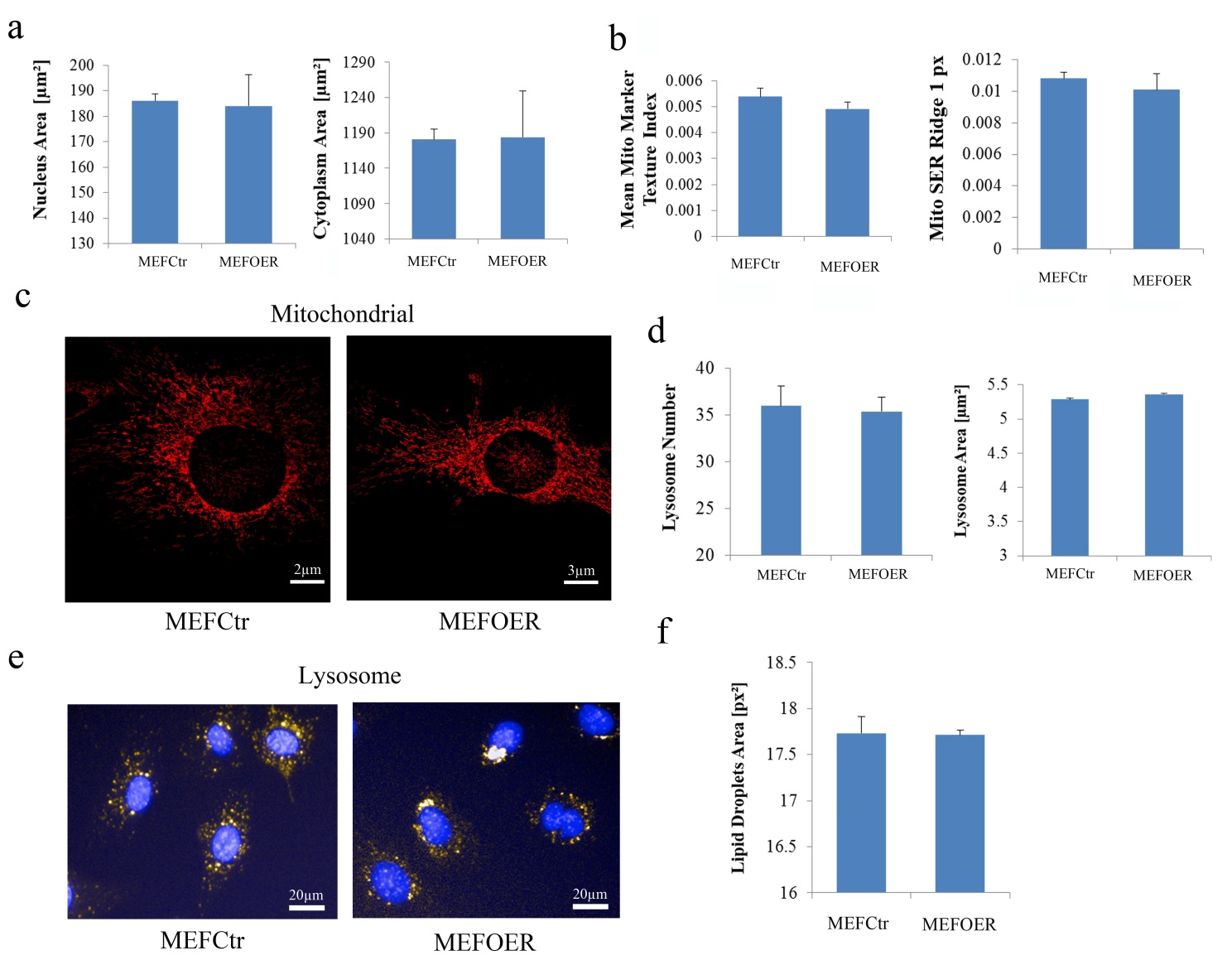


**Supplementary Figure 5. Overexpression of Rab32 in MEFs did not affect the cell morphology, lysosomes and mitochondrial.**

(**a**) The nucleus and cytoplasm area did not produce marked changes after overexpression of Rab32 in MEFs.

(**b** and **c**) The intensity structure of mitochondrial which was analyzed for the occurrence of typical intensity patterns, did not change as the Rab32 level increased (b), and the representative pictures were shown in c (bar = 2 µm, left; bar = 3 µm, right).

(**d and e**) Extra-expression of Rab32 did not affect the lysosomes number and area (bar = 20 µm). The results of MEFCtr were used as control.

(**f**) Extra-expression of Rab32 had no influence on average area of lipid droplets compared to the control (n = 3, *p < 0.05). The results of MEFCtr were used as control.


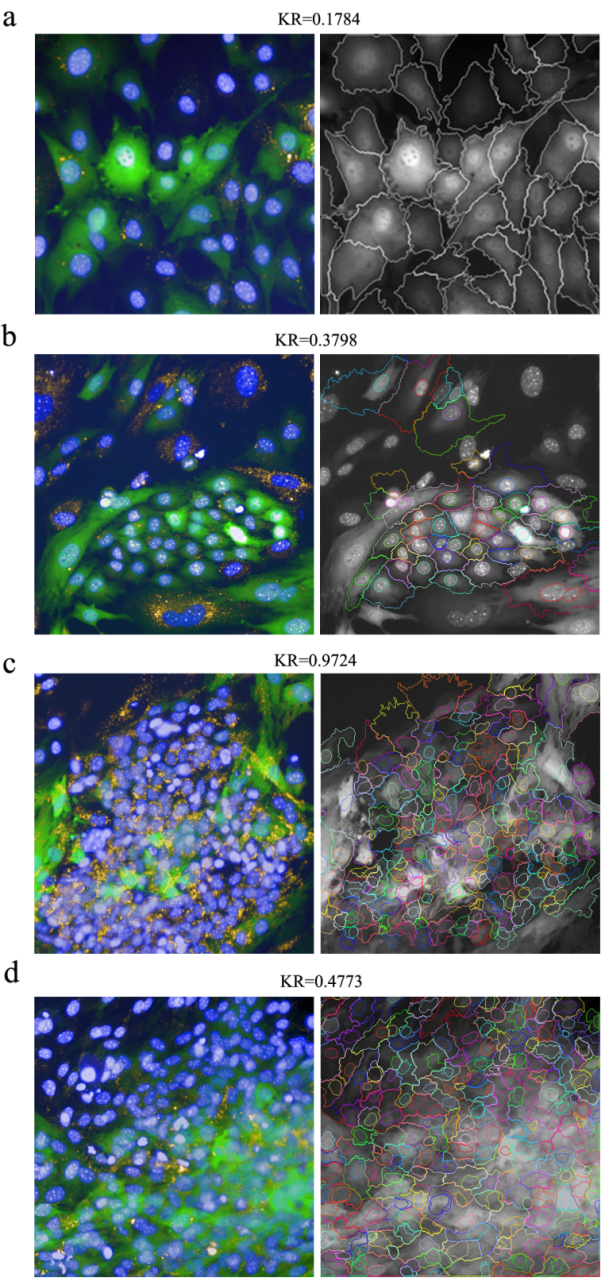
**Supplementary Figure 6. Images of reprogramming cells and the changes of morphology and KR**

Pluripotent stem cells are smaller and have a higher karyoplasmic ratio then differentiated ones. We used Harmony software to analyze nuclear area and cytoplasm area, then calculated KR= nuclear area / cytoplasm area.

(**a**) The cells morphology after being seeded on plate one day later. The average of KR was 0.18.

(**b**) The cells morphology during early stage of iPSC induction. The average of KR was 0.38.

(**c**) The cells morphology during middle stage of iPSC induction. The average of KR was 0.97.

(**d**) The cells morphology during later stage of iPSC induction. Because the cells were too close to each other, and some were overlapped, the software could not accurately distinguish each cell during late stage of reprogramming.

So, we only used Harmony software to analyze the full-scan pictures of early and middle stages (d1 to d8, KR > 0.18). The average KR in A, B, C and D were calculated from 20 specific fields.

**
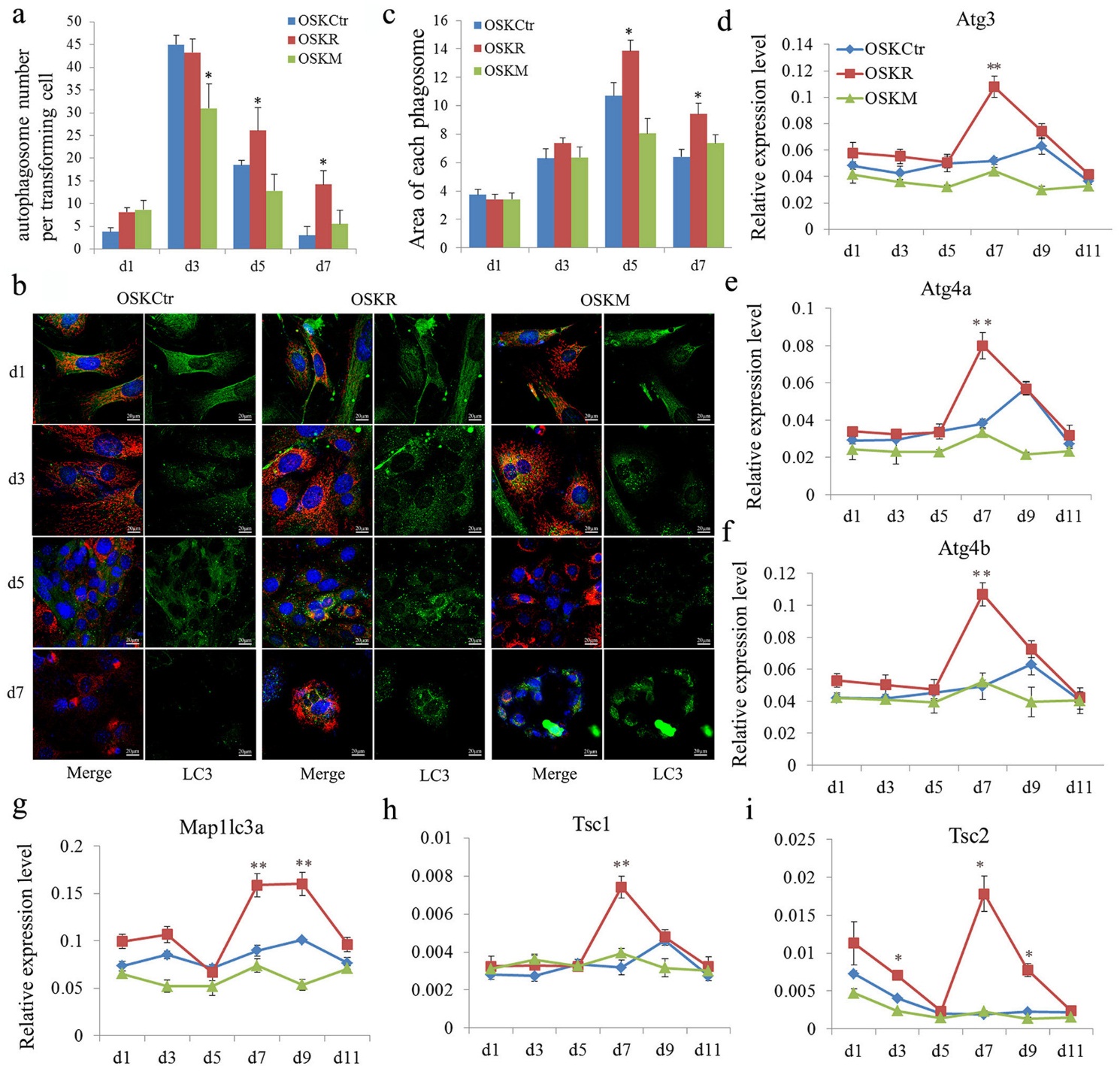
** **Supplementary Figure 7. Rab32 enhances autophagy during reprogramming**

(**a**) Rab32 activated autophagy during reprogramming. LC3^+^ dots per cell were calculated and compared to the MEF inducted with OSKCtr on the same day (n = 3, *p < 0.05, the results of OSKCtr were used as control, *p < 0.05).

(**b**) The representative images showed the changes of phagosomes during reprogramming (d1,d3, d5, d7). Autophagosomes were stained with LC3 antibody and the nuclei were stained with DAPI in mitochondrial-RFP cells seeded on feeder (bar = 50 µm).

(**c**) Autophagosome area per dot was calculated and compared with the MEF induction with OSKCtr on the same day (n = 3, the results of OSKCtr were used as control, *p < 0.05, ** p < 0.01).

(**d, e, f, h and i**) The expression levels of Atg3, Atg4a, Atg4b, Mapllc3a, Tsc1, Tsc2 were determined by qPCR on day 1, 3, 5, 7, 9 and 11 during reprogramming with OSKCtr, OSKR and OSKM. The results of OSKCtr were used as control. *p < 0.05, ** p < 0.01.


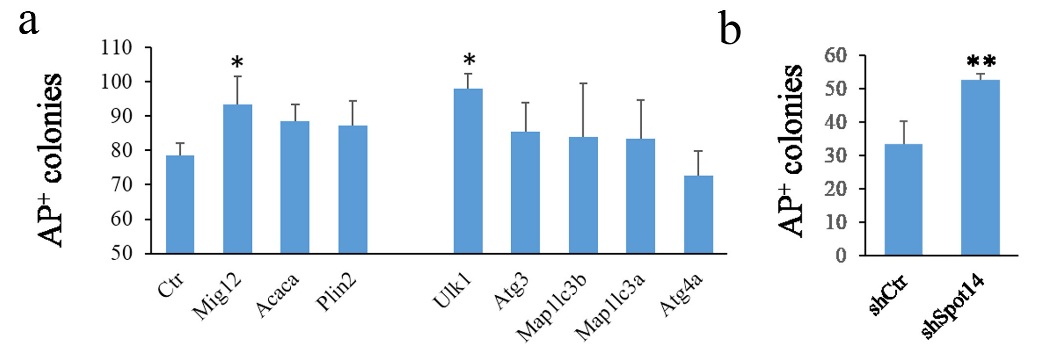


**Supplementary Figure 8. Reprogramming efficiency increased by overexpression of Mig12 and Ulk1, and by knockdown of Spot14 in** **OG2 MEFs transfected with OSK.**

1. Quantification of AP^+^ colony numbers after overexpression of OSK plus one of genes regulating autophagy and lipid metabolism. The results of Ctr (OG2 MEFs transfected with OSK and pMXs empty retroviral vector) were used as control. n=3, *p < 0.05).
2. AP^+^ colony numbers were affected by knockdown Spot14 in OG2 MEFs transfected with OSK. The results of shCtr (OG2 MEFs transfected with OSK and shRNA negative control) were used as control. n=3, *p < 0.05, **p < 0.01.

**Supplementary Table 1. Relative expression quantity of lipid metabolism related genes** during reprogramming

|  |  | Day 1 | Day 3 | Day 5 | Day 7 | Day 9 | Day 11 |
| --- | --- | --- | --- | --- | --- | --- | --- |
| Acaca | OSKCtr | 8.9±0.2×10^-3^(A) | 9.1±0.4×10^-3^(A) | 1.1±0.1×10^-2^(A) | 8.8±0.1×10^-3^(A) | 1.1±0.1×10^-2^(A) | 7.8±0.2×10^-3^ |
|  | OSKR | 1.2±0.4×10^-2^(a, B) | 1.1±0.2×10^-2^(a, A) | 1.3±0.3×10^-2^(a, B) | 1.4±0.2×10^-2^(a, B) | 1.0±0.3×10^-2^(ab, A) | 6.7±0.2×10^-3^(b) |
|  | OSKM | 1.4±0.6×10^-2^(a, C) | 1.4±0.1×10^-2^(a, B) | 1.4±0.3×10^-2^(a, B) | 1.2±0.2×10^-2^(ab, C) | 7.1±0.9×10^-3^(b, B) | 6.7±0.4×10^-3^(b) |
| Acacb | OSKCtr | 6.8±0.3×10^-4^(a) | 7.3±0.1×10^-4^(a) | 2.1±0.6×10^-3^(b) | 2.7±0.3×10^-3^(b) | 4.8±0.2×10^-3^(c, A) | 2.0±0.4×10^-3^(b, A) |
|  | OSKR | 7.2±0.5×10^-4^(a) | 9.4±0.4×10^-4^(a) | 1.7±0.2×10^-3^(b) | 2.5±0.3×10^-3^(b) | 3.6±0.5×10^-3^(c, B) | 3.4±0.8×10^-3^(c, B) |
|  | OSKM | 1.3±0.4×10^-3^ | 2.1±0.2×10^-3^ | 1.4±0.3×10^-3^ | 2.5±0.2×10^-3^ | 2.1±0.4×10^-3^(C) | 1.3±0.4×10^-3^(A) |
| Etfdh | OSKCtr | 4.8±0.2×10^-2^(a) | 5.2±0.4×10^-2^(a) | 7.3±0.5×10^-2^(a) | 7.9±0.1×10^-2^(a) | 1.0±0.1×10^-1^(b, C) | 6.2±0.4×10^-2^(a, B) |
|  | OSKR | 5.2±0.3×10^-2^(a) | 5.4±0.5×10^-2^(a) | 6.8±0.3×10^-2^(a) | 1.3±0.3×10^-1^(b) | 1.0±0.1×10^-1^(bc, B) | 7.3±0.5×10^-2^(a, B) |
|  | OSKM | 5.5±0.4×10^-2^(a) | 6.2±0.2×10^-2^(a) | 8.1±0.5×10^-2^(a) | 1.1±0.1×10^-1^(b) | 6.4±0.4×10^-2^(a, A) | 8.0±0.6×10^-2^(a, A) |
| Fasn | OSKCtr | 2.2±0.3×10^-2^(A) | 1.8±0.2×10^-2^(A) | 2.2±0.1×10^-2^ | 1.8±0.2×10^-2^ | 2.5±0.3×10^-2^ | 1.4±0.2×10^-2^ |
|  | OSKR | 4.2±0.4×10^-2^(a, B) | 3.0±0.2×10^-2^(a, B) | 3.4±0.3×10^-2^(a) | 3.1±0.2×10^-2^(ab) | 2.7±0.1×10^-2^(ab) | 1.9±0.3×10^-2^(b) |
|  | OSKM | 4.7±0.2×10^-2^(B) | 4.5±0.4×10^-2^(A) | 4.4±0.2×10^-2^ | 3.5±0.3×10^-2^ | 2.6±0.2×10^-2^ | 2.3±0.2×10^-2^ |
| Lipin1 | OSKCtr | 1.5±0.4×10^-3^ | 1.9±0.1×10^-3^ | 2.0±0.3×10^-3^ | 2.0±0.4×10^-3^(C) | 2.7±0.1×10^-3^(A) | 2.0±0.3×10^-3^(B) |
|  | OSKR | 1.5±0.2×10^-3^(a) | 2.9±0.3×10^-3^(a) | 1.8±0.5×10^-3^(a) | 1.2±0.5×10^-2^(b, B) | 6.1±0.3×10^-3^(c, B) | 2.4±0.1×10^-3^(a, B) |
|  | OSKM | 2.2±0.1×10^-3^ | 5.1±0.2×10^-3^ | 3.0±0.1×10^-3^ | 6.6±0.3×10^-3^(A) | 3.6±0.2×10^-3^(A) | 5.0±0.2×10^-3^(A) |
| Hmgcs | OSKCtr | 8.4±0.4×10^-5^(a) | 6.1±0.2×10^-5^(a) | 3.9±0.5×10^-3^(b, A) | 4.4±0.2×10^-3^(bc, A) | 5.4±0.1×10^-3^(c, B) | 2.5±0.3×10^-3^(bd, B) |
|  | OSKR | 8.6±0.6×10^-5^(a) | 4.4±0.1×10^-5^(a) | 2.2±0.1×10^-3^(b, B) | 7.7±0.3×10^-4^(a, B) | 2.3±0.2×10^-3^(b, A) | 3.3±0.1×10^-4^(bc, B) |
|  | OSKM | 4.4±0.2×10^-5^(a) | 6.3±0.3×10^-5^(a) | 5.3±0.7×10^-3^(b, A) | 3.9±0.7×10^-3^(bc, A) | 2.5±0.3×10^-3^(d, A) | 5.3±0.6×10^-4^(a, A) |
| Mlycd | OSKCtr | 9.7±0.5×10^-3^(a) | 8.9±0.2×10^-3^(a) | 1.2±0.1×10^-3^(a) | 1.6±0.1×10^-2^(b, A) | 2.2±0.2×10^-2^(c, B) | 1.1±0.3×10^-2^(ab) |
|  | OSKR | 1.1±0.2×10^-2^(a) | 9.1±0.3×10^-3^(a) | 1.2±0.4×10^-2^(a) | 2.3±0.7×10^-2^(b, B) | 2.0±0.3×10^-2^(b, B) | 1.5±0.1×10^-2^(ac) |
|  | OSKM | 9.9±0.3×10^-3^(a) | 9.2±0.1×10^-3^(a) | 1.3±0.3×10^-2^(a) | 1.9±0.1×10^-2^(b, A) | 1.3±0.4×10^-2^(a, A) | 1.4±0.2×10^-2^(a) |
| Mig12 | OSKCtr | 7.4±0.2×10^-3^(a, A) | 5.0±0.2×10^-3^(b) | 8.3±0.2×10^-3^(a, B) | 6.5±0.2×10^-3^(ab, A) | 1.0±0.2×10^-2^(c, B) | 4.9±0.2×10^-3^(ab, AB) |
|  | OSKR | 1.0±0.1×10^-2^(a, B) | 7.1±0.3×10^-3^(b) | 9.1±1.0×10^-3^(ab, B) | 1.2±0.1×10^-2^(c, B) | 9.8±0.1×10^-3^(ab, B) | 7.5±0.4×10^-3^(b, B) |
|  | OSKM | 7.8±0.3×10^-3^(a, A) | 6.3±0.2×10^-3^(a) | 5.3±0.8×10^-3^(a, A) | 5.2±0.4×10^-3^(a, A) | 4.3±0.3×10^-3^(ab, A) | 2.9±0.3×10^-3^(b, A) |
| Plin2 | OSKCtr | 1.9±0.2×10^-1^ | 1.8±0.1×10^-1^ | 1.7±0.2×10^-1^ | 1.9±0.2×10^-1^(C) | 2.9±0.3×10^-1^(A) | 2.0±0.1×10^-1^(B) |
|  | OSKR | 1.5±0.1×10^-1^(a) | 1.7±0.4×10^-1^(a) | 1.5±0.1×10^-1^(a) | 9.2±0.1×10^-1^(b, B) | 4.9±0.6×10^-1^(c, B) | 2.2±0.2×10^-1^(a, B) |
|  | OSKM | 1.6±0.3×10^-1^ | 2.0±0.2×10^-1^ | 2.4±0.3×10^-1^ | 4.2±0.3×10^-1^(A) | 3.3±0.2×10^-1^(A) | 5.1±0.4×10^-1^(A) |
| Plin3 | OSKCtr | 1.5±0.1×10^-2^(a) | 1.8±0.1×10^-2^(a) | 2.6±0.2×10^-2^(a, AB) | 3.4±0.2×10^-2^(a, A) | 4.6±0.3×10^-2^(b, B) | 2.7±0.2×10^-2^(a, A) |
|  | OSKR | 2.0±0.4×10^-2^(a) | 2.8±0.1×10^-2^(a) | 3.4±0.7×10^-2^(ac, B) | 6.1±0.4×10^-2^(b, B) | 5.4±0.4×10^-2^(bc, B) | 4.3±0.3×10^-2^(c, B) |
|  | OSKM | 1.6±0.1×10^-2^ | 1.5±0.2×10^-2^ | 1.8±0.1×10^-2^(A) | 2.9±0.3×10^-2^(A) | 2.4±0.2×10^-2^(A) | 2.5±0.3×10^-2^(A) |
| Spot14 | OSKCtr | 3.0±0.7×10^-4^(a) | 1.9±0.1×10^-4^(b, B) | 3.1±0.6×10^-4^(ab, C) | 4.1±0.2×10^-4^(a, C) | 6.9±0.5×10^-4^(c, B) | 3.6±0.5×10^-4^(a, C) |
|  | OSKR | 3.5±0.2×10^-4^(a) | 5.9±0.1×10^-4^(b, A) | 1.5±0.5×10^-4^(b, B) | 1.6±0.3×10^-4^(b, B) | 2.7±0.2×10^-4^(ab, A) | 1.0±0.4×10^-3^(c, B) |
|  | OSKM | 4.6±0.3×10^-4^(a) | 3.2±0.2×10^-4^(b, A) | 1.1±0.1×10^-3^(c, A) | 7.6±0.3×10^-4^(d, A) | 5.6±0.4×10^-4^(ab, A) | 6.1±0.2×10^-4^(ab, A) |

The gene expression profiles were expressed using the comparative CT (2^-ΔCT^) method. The ΔCT was calculated using β-actin, EF1-α, and α-tubulin as internal control. Data were shown as M ± SD, n=3. The mean values of each group were analyzed by Duncan's multiple range tests. Small letters mean significant at 0.05 level compared by the means at the same row, and capital letters mean significant at 0.05 level compared the means in the same column.

**Supplementary Table 2. Relative expression quantity of autophagy-related genes during reprogramming**

|  |  | Day 1 | Day 3 | Day 5 | Day 7 | Day 9 | Day 11 |
| --- | --- | --- | --- | --- | --- | --- | --- |
| Atg3 | OSKCtr | 4.8±0.3×10^-2^ | 4.3±0.5×10^-2^ | 5.0±0.6×10^-2^ | 5.2±0.3×10^-2^(A) | 6.3±0.7×10^-2^(A) | 3.6±0.5×10^-2^ |
|  | OSKR | 5.8±0.8×10^-2^(a) | 5.5.±0.4×10^-2^(a) | 5.0±0.3×10^-2^(a) | 1.1±0.1×10^-1^(b, B) | 7.4±0.6×10^-2^(ab, A) | 4.1±0.3×10^-2^(ac) |
|  | OSKM | 4.2±0.6×10^-2^ | 3.6±0.2×10^-2^ | 3.2±0.1×10^-2^ | 4.4±0.2×10^-2^(A) | 3.0±0.2×10^-2^(B) | 3.2±0.1×10^-2^ |
| Atg4a | OSKCtr | 2.9±0.2×10^-2^(a) | 2.9±0.3×10^-2^(a) | 3.4±0.4×10^-2^(a) | 3.8±0.2×10^-2^(a) | 5.7±0.3×10^-2^(b, A) | 2.7±0.2×10^-2^(a) |
|  | OSKR | 3.4±0.2×10^-2^(a) | 3.2±0.2×10^-2^(a) | 3.3±0.2×10^-2^(a) | 8.0±0.7×10^-2^(b) | 5.7±0.4×10^-2^(c, A) | 3.2±0.5×10^-2^(a) |
|  | OSKM | 2.4±0.5×10^-2^ | 2.3±0.6×10^-2^ | 2.3±0.1×10^-2^ | 3.3±0.2×10^-2^ | 2.1±0.1×10^-2^(B) | 2.3±0.1×10^-2^ |
| Atg4b | OSKCtr | 4.2±0.3×10^-2^ | 4.2±0.2×10^-2^ | 4.5±0.4×10^-2^ | 4.9±0.5×10^-2^(A) | 6.3±0.7×10^-2^(A) | 4.0±0.8×10^-2^ |
|  | OSKR | 5.3±0.4×10^-2^(a) | 5.0.±0.6×10^-2^(a) | 4.7±0.6×10^-2^(a) | 1.1±0.3×10^-1^(b, B) | 7.2±0.5×10^-2^(c) | 4.2±0.3×10^-2^(a) |
|  | OSKM | 4.2±0.2×10^-2^ | 4.1±0.1×10^-2^ | 3.9±0.7×10^-2^ | 5.2±0.1×10^-2^(A) | 4.0±0.1×10^-2^(B) | 4.1±0.5×10^-2^ |
| Akt | OSKCtr | 8.8±0.6×10^-2^(A) | 1.0±0.3×10^-1^(A) | 1.0±0.1×10^-1^(A) | 9.8±0.7×10^-2^ | 1.0±0.2×10^-1^ | 8.8±0.6×10^-2^ |
|  | OSKR | 1.1±0.4×10^-1^(a, AB) | 1.0±0.2×10^-1^(a, A) | 1.2±0.3×10^-1^(a, A) | 1.6±0.2×10^-1^ | 1.1±0.4×10^-1^ | 1.1±0.4×10^-1^ |
|  | OSKM | 7.1±0.6×10^-2^(B) | 6.0±0.5×10^-2^(B) | 6.3±0.5×10^-2^(B) | 6.3±0.8×10^-2^ | 4.8±0.5×10^-2^ | 7.1±0.6×10^-2^ |
| Lkb1 | OSKCtr | 9.7±0.8×10^-2^(a, B) | 8.2±0.7×10^-2^(a, B) | 1.0±0.1×10^-1^(a, B) | 1.2±0.1×10^-1^(a, B) | 1.7±0.2×10^-1^(b, AB) | 9.0±0.7×10^-2^(a) |
|  | OSKR | 1.4±0.1×10^-1^(a, B) | 1.2±0.2×10^-1^(a, B) | 1.3±0.1×10^-1^(a, B) | 3.1±0.2×10^-1^(b, B) | 2.3±0.1×10^-1^(c, B) | 1.3±0.1×10^-1^(a) |
|  | OSKM | 9.4±0.5×10^-2^(a, A) | 8.8±0.6×10^-2^(a, A) | 9.1±0.7×10^-2^(a, A) | 1.4±0.3×10^-1^(b, A) | 9.2±0.8×10^-2^(a, A) | 1.1±0.2×10^-1^(a) |
| Redd1 | OSKCtr | 1.2±0.2×10^-4^(a) | 2.2±0.2×10^-4^(a) | 3.9±0.5×10^-4^(a, C) | 1.2±0.2×10^-3^(b, B) | 4.8±0.3×10^-4^(a) | 2.0±0.1×10^-4^(a) |
|  | OSKR | 1.8±0.4×10^-4^(a) | 1.9±0.3×10^-4^(a) | 1.1±0.1×10^-3^(b, B) | 2.2±0.3×10^-4^(a, A) | 7.1±0.4×10^-4^(c) | 3.7±0.4×10^-4^(a) |
|  | OSKM | 4.3±0.3×10^-4^(a) | 5.9±0.6×10^-4^(a) | 2.7±0.3×10^-3^(b, A) | 3.0±0.5×10^-4^(c, A) | 5.0±0.7×10^-4^(ac) | 2.7±0.2×10^-4^(ac) |
| Ulk1 | OSKCtr | 2.4±0.2×10^-4^(a,A) | 3.7±0.4×10^-4^(b) | 5.9±0.6×10^-4^(c,A) | 6.8±0.2×10^-4^(d,AB) | 8.8±0.6×10^-4^(e,A) | 4.2±0.3×10^-4^(c,A) |
|  | OSKR | 1.9±0.3×10^-4^(a,A) | 3.7±0.6×10^-4^(b) | 5.7±0.4×10^-4^(c,A) | 7.9±0.5×10^-4^(d,A) | 9.6±0.3×10^-4^(e,A) | 5.2±0.4×10^-4^(c,A) |
|  | OSKM | 4.0±0.2×10^-4^(a,B) | 4.1±0.3×10^-4^(a) | 4.3±0.5×10^-4^(a,B) | 5.7±0.4×10^-4^(b,B) | 6.2±0.8×10^-4^(bc,B) | 7.2±0.9×10^-4^(c,B) |
| Map1lc3a | OSKCtr | 7.3±0.5×10^-2^(a, A) | 8.5±0.7×10^-2^(ab, AB) | 7.1±0.5×10^-2^(a) | 9.0±0.7×10^-2^(ab, A) | 1.0±0.5×10^-1^(b, C) | 7.6±0.5×10^-2^(a, A) |
|  | OSKR | 9.9±0.7×10^-2^(a, B) | 1.0±0.1×10^-1^(b, B) | 6.6±0.8×10^-2^(b) | 1.5±0.1×10^-1^(c, B) | 1.6±0.1×10^-1^(c, B) | 9.6±0.8×10^-2^(a, B) |
|  | OSKM | 6.5±0.5×10^-2^(A) | 5.2±0.4×10^-2^(A) | 5.2±0.4×10^-2^ | 7.4±0.4×10^-2^(A) | 5.4±0.2×10^-2^(A) | 7.1±0.6×10^-2^(A) |
| Map1lc3b | OSKCtr | 1.9±0.3×10^-1^(a, AB) | 2.2±0.4×10^-1^(a, AB) | 2.6±0.2×10^-1^(a) | 3.0±0.2×10^-1^(ab, A) | 4.5±0.3×10^-1^(b, B) | 2.8±0.1×10^-1^(a) |
|  | OSKR | 2.2±0.3×10^-1^(a, B) | 2.9±0.2×10^-1^(a, B) | 2.3±0.4×10^-1^(a) | 6.5±0.5×10^-1^(b, B) | 4.8±0.4×10^-1^(c, B) | 2.8±0.2×10^-1^(a) |
|  | OSKM | 1.4±0.1×10^-1^(a, A) | 1.7±0.3×10^-1^(a, A) | 1.9±0.2×10^-1^(a) | 3.5±0.3×10^-1^(b, A) | 2.3±0.2×10^-1^(a, A) | 3.0±0.4×10^-1^(ab) |

The gene expression profiles were expressed using the comparative CT (2^-ΔCT^) method. The ΔCT was calculated using β-actin, EF1-α and α-tubulin as internal controls. Data were shown as M ± SD, n=3. The mean values of each group were analyzed by Duncan's multiple range tests. Small letters means significant at 0.05 level compared by the means at the same row, and capital letters means significant at 0.05 level compared by the means at the same column.

**Supplementary Table 3. Relative expression quantity of key genes in specific pathways** during reprogramming

|  |  | d1 | d3 | d5 | d7 | d9 | d11 |
| --- | --- | --- | --- | --- | --- | --- | --- |
| Tsc1 | OSKCtr | 2.8±0.1×10^-3^(a) | 2.7±0.2×10^-3^(a) | 3.4±0.3×10^-3^(a) | 3.2±0.3×10^-3^(a, A) | 4.6±0.5×10^-3^(b, B) | 2.7±0.2×10^-3^(a) |
|  | OSKR | 3.2±0.5×10^-3^(a) | 3.3±0.6×10^-3^(a) | 3.2±0.3×10^-3^(a) | 7.4±0.5×10^-3^(b, B) | 4.8±0.4×10^-3^(c, B) | 3.2±0.5×10^-3^(a) |
|  | OSKM | 3.1±0.2×10^-3^ | 3.6±0.3×10^-3^ | 3.3±0.2×10^-3^ | 3.9±0.4×10^-3(A)^ | 3.2±0.2×10^-3(A)^ | 3.0±0.2×10^-3^ |
| Tsc2 | OSKCtr | 7.3±0.6×10^-3^(a, AB) | 4.0±0.1×10^-3^(ab, A) | 2.0±0.2×10^-3^(b) | 1.9±0.6×10^-3^(b, A) | 2.2±0.2×10^-3^(b, A) | 2.2±0.3×10^-3^(b) |
|  | OSKR | 1.1±0.3×10^-2^(a, B) | 7.1±0.5×10^-3^(b, AB) | 2.4±0.3×10^-3^(c) | 1.8±0.2×10^-2^(d, B) | 7.8±0.9×10^-3^(b, B) | 2.4±0.2×10^-3^(c) |
|  | OSKM | 4.7±0.2×10^-3^(a, A) | 2.4±0.2×10^-3^(ab, A) | 1.4±0.1×10^-3^(b) | 2.3±0.3×10^-3^(b, A) | 1.3±0.1×10^-3^(b, A) | 1.5±0.3×10^-3^(b) |
| Mtor | OSKCtr | 3.5±0.9×10^-4^ | 7.1±0.9×10^-5^ | 4.3±1.0×10^-4^ | 2.7±0.2×10^-4^ | 2.7±0.4×10^-4^ | 8.1±0.5×10^-5^ |
|  | OSKR | 9.2±1.7×10^-5^ | 7.7±0.6×10^-5^ | 9.0±0.6×10^-5^ | 7.5±1.3×10^-5^ | 7.7±1.1×10^-5^ | 9.6±1.4×10^-5^ |
|  | OSKM | 1.1±0.3×10^-4^ | 8.9±0.4×10^-5^ | 1.2±0.3×10^-4^ | 1.4±0.5×10^-4^ | 1.7±0.2×10^-4^ | 1.5±0.3×10^-4^ |
| AMPKa1 | OSKCtr | 1.3±0.3×10^-1^ | 6.7±0.5×10^-2^ | 5.6±0.5×10^-2^ | 4.9±0.5×10^-2^ | 7.0±0.4×10^-2^ | 4.2±0.7×10^-2^ |
|  | OSKR | 6.9±0.5×10^-2^ | 8.4±0.7×10^-2^ | 7.5±0.6×10^-2^ | 6.7±0.6×10^-2^ | 1.1±0.6×10^-1^ | 6.2±0.5×10^-2^ |
|  | OSKM | 6.4±0.4×10^-2^ | 7.8±0.7×10^-2^ | 5.9±0.6×10^-2^ | 5.9±0.7×10^-2^ | 5.6±0.7×10^-2^ | 7.3±1.5×10^-2^ |
| Notch1 | OSKCtr | 1.7±0.2×10^-2^ | 1.3±0.3×10^-2^ | 9.5±1.5×10^-3^ | 1.2±0.4×10^-2^ | 1.1±0.4×10^-2^ | 2.2±0.4×10^-2^ |
|  | OSKR | 5.9±0.2×10^-3^ | 2.2±0.3×10^-2^ | 1.3±0.2×10^-2^ | 1.4±0.3×10^-2^ | 2.2±0.1×10^-2^ | 2.5±0.4×10^-2^ |
|  | OSKM | 1.0±0.4×10^-2^ | 1.6±0.4×10^-2^ | 1.5±0.3×10^-2^ | 8.7±0.5×10^-3^ | 1.2±0.2×10^-2^ | 7.3±0.6×10^-3^ |
| Stat3 | OSKCtr | 2.2±0.1×10^-1^ | 9.5±1.2×10^-2^ | 1.1±0.4×10^-1^ | 1.3±0.3×10^-1^ | 1.5±0.2×10^-1^ | 2.1±0.2×10^-1^ |
|  | OSKR | 1.2±0.2×10^-1^ | 1.1±0.2×10^-1^ | 1.2±0.3×10^-1^ | 1.3±0.4×10^-1^ | 2.0±0.1×10^-1^ | 2.1±0.3×10^-1^ |
|  | OSKM | 1.0±0.3×10^-1^ | 1.0±0.3×10^-1^ | 1.2±0.3×10^-1^ | 1.1±0.5×10^-1^ | 1.3±0.2×10^-1^ | 9.4±1.3×10^-2^ |
| GSK3b | OSKCtr | 9.4±0.6×10^-2^ | 6.4±1.2×10^-2^ | 7.3±0.3×10^-2^ | 1.0±0.1×10^-1^ | 8.2±1.0×10^-2^ | 1.2±0.1×10^-1^ |
|  | OSKR | 8.6±0.8×10^-2^ | 7.8±0.6×10^-2^ | 8.2±1.1×10^-2^ | 9.7±1.4×10^-2^ | 1.4±0.2×10^-1^ | 1.1±0.3×10^-1^ |
|  | OSKM | 8.0±0.5×10^-2^ | 6.3±1.4×10^-2^ | 8.3±0.6×10^-2^ | 8.2±0.6×10^-2^ | 8.4±1.3×10^-2^ | 7.4±1.1×10^-2^ |
| PI3K | OSKCtr | 5.8±0.4×10^-4^ | 1.6±0.2×10^-5^ | 1.2±0.4×10^-5^ | 4.4±0.6×10^-4^ | 4.4±0.8×10^-4^ | 2.7±0.3×10^-3^ |
|  | OSKR | 1.2±0.5×10^-4^ | 1.4±0.2×10^-5^ | 2.8±0.3×10^-5^ | 8.3±1.2×10^-5^ | 8.3±0.4×10^-5^ | 4.2±0.4×10^-4^ |
|  | OSKM | 2.6±0.7×10^-3^ | 4.3±0.4×10^-5^ | 8.4±1.2×10^-5^ | 2.5±0.5×10^-4^ | 2.5±0.3×10^-4^ | 4.6±0.5×10^-5^ |
| Nodal | OSKCtr | 6.1±0.3×10^-4^ | 3.4±0.8×10^-4^ | 2.0±0.4×10^-4^ | 3.9±0.4×10^-4^ | 5.3±1.1×10^-4^ | 5.5±1.2×10^-3^ |
|  | OSKR | 3.9±0.9×10^-3^ | 4.6±0.9×10^-4^ | 2.2±0.2×10^-4^ | 2.2±0.3×10^-4^ | 4.7±0.3×10^-4^ | 3.7±0.3×10^-3^ |
|  | OSKM | 7.7±1.2×10^-4^ | 7.7±1.8×10^-4^ | 2.9±0.3×10^-4^ | 6.5±0.7×10^-4^ | 2.4±0.4×10^-3^ | 4.2±0.9×10^-3^ |
| Bmp4 | OSKCtr | 1.1±0.3×10^-2^ | 1.3±0.5×10^-2^ | 9.5±1.3×10^-3^ | 1.1±0.3×10^-2^ | 1.2±0.5×10^-2^ | 1.2±0.4×10^-2^ |
|  | OSKR | 1.2±0.2×10^-2^ | 1.5±0.3×10^-2^ | 8.2±0.7×10^-3^ | 1.6±0.4×10^-2^ | 9.9±0.7×10^-3^ | 9.0±0.8×10^-3^ |
|  | OSKM | 8.7±0.9×10^-3^ | 6.7±0.7×10^-3^ | 5.9±0.9×10^-3^ | 1.1±0.2×10^-2^ | 5.5±0.7×10^-3^ | 7.2±0.7×10^-3^ |
| Erk2 | OSKCtr | 5.2±0.4×10^-2^(a, C) | 7.3±0.9×10^-2^(a, B) | 9.1±0.1×10^-2^(a, B) | 9.1±0.2×10^-1^(ab, C) | 1.5±0.3×10^-1^(b, B) | 4.0±0.4×10^-2^(a, B) |
|  | OSKR | 6.2±0.9×10^-2^(a, B) | 1.0±0.1×10^-1^(b, B) | 1.0±0.3×10^-1^(b, B) | 1.7±0.2×10^-1^(c, B) | 1.5±0.2×10^-1^(d, B) | 6.3±0.3×10^-2^(a, AB) |
|  | OSKM | 3.6±0.3×10^-2^(a, A) | 6.3±0.7×10^-2^(a, A) | 6.1±0.4×10^-2^(a, A) | 9.1±0.6×10^-2^(b, A) | 4.6±0.3×10^-2^(a, A) | 4.3±0.1×10^-2^(a, A) |

The gene expression profiles were expressed using the comparative CT (2^-ΔCT^) method. The ΔCT was calculated using β-actin, EF1-α, and α-tubulin as internal control. Data were shown as M ± SD, n=3. The mean values of each group were analyzed by Duncan's multiple range tests. Small letters means significant at 0.05 level compared by the means at the same row, and capital letters means significant at 0.05 level compared the means in the same column.

**Supplementary Table 4. Relative expression quantity of transgenes and pluripotency genes during** **reprogramming.**

|  |  | d1 | d3 | d5 | d7 | d9 | d11 |
| --- | --- | --- | --- | --- | --- | --- | --- |
| ex-Oct4 | OSKCtr | 9.7±1.7×10^-2^ | 1.0±0.3×10^-1^ | 4.1±0.1×10^-1^ | 2.9±0.4×10^-1^ | 3.1±0.8×10^-1^ | 1.7±0.1×10^-1^ |
|  | OSKR | 1.4±0.3×10^-1^ | 2.3±0.3×10^-1^ | 3.0±0.5×10^-1^ | 3.4±0.5×10^-1^ | 3.4±0.4×10^-1^ | 2.4±0.2×10^-1^ |
|  | OSKM | 1.5±0.1×10^-1^ | 2.1±0.3×10^-1^ | 3.7±0.1×10^-1^ | 3.5±0.4×10^-1^ | 2.4±0.6×10^-1^ | 2.9±0.3×10^-1^ |
| ex-Sox2 | OSKCtr | 9.6±0.6×10^-2^ | 1.5±0.2×10^-1^ | 2.0±0.2×10^-1^ | 2.3±0.4×10^-1^ | 2.9±0.5×10^-1^ | 2.2±0.4×10^-1^ |
|  | OSKR | 6.5±0.2×10^-2^ | 9.5±2.9×10^-2^ | 1.4±0.4×10^-1^ | 1.6±0.3×10^-1^ | 1.9±0.3×10^-1^ | 1.5±0.5×10^-1^ |
|  | OSKM | 1.7±0.5×10^-1^ | 1.7±0.2×10^-1^ | 2.1±0.4×10^-1^ | 1.6±0.3×10^-1^ | 1.4±0.5×10^-1^ | 1.1±0.4×10^-1^ |
| ex-Klf4 | OSKCtr | 3.7±0.5×10^-2^ | 8.0±0.3×10^-2^ | 1.3±0.2×10^-1^ | 1.3±0.2×10^-1^ | 1.2±0.2×10^-1^ | 1.0±0.2×10^-1^ |
|  | OSKR | 1.5±0.7×10^-2^ | 4.6±0.6×10^-2^ | 1.1±0.6×10^-1^ | 7.2±0.6×10^-2^ | 1.3±0.3×10^-1^ | 8.2±0.6×10^-2^ |
|  | OSKM | 5.7±0.8×10^-2^ | 6.2±0.7×10^-2^ | 1.2±0.1×10^-1^ | 6.4±0.4×10^-2^ | 4.7±0.5×10^-2^ | 4.1±0.3×10^-2^ |
| ex-c-Myc | OSKCtr | 0 | 0 | 0 | 0 | 0 | 0 |
|  | OSKR | 0 | 0 | 0 | 0 | 0 | 0 |
|  | OSKM | 8.6±0.8×10^-1^ | 1.0±0.2 | 1.3±0.5 | 1.2±0.3×10^-3^ | 9.5±1.1×10^-1^ | 9.6±0.8×10^-1^ |
| ex-Rab32 | OSKCtr | 0 | 0 | 0 | 0 | 0 | 0 |
|  | OSKR | 8.3±0.5×10^-3^ | 1.9±0.8×10^-2^ | 4.7±0.9×10^-2^ | 4.5±0.7×10^-2^ | 1.1±0.2×10^-1^ | 5.1±0.8×10^-2^ |
|  | OSKM | 0 | 0 | 0 | 0 | 0 | 0 |
| en-Oct4 | OSKCtr | 1.1±0.2×10^-3^ | 2.2±0.8×10^-3^ | 2.1±0.6×10^-3^ | 3.1±1.0×10^-3^ | 1.8±0.6×10^-3^ | 2.5±0.6×10^-3^ |
|  | OSKR | 1.5±0.2×10^-3^ | 9.6±0.6×10^-4^ | 1.8±0.5×10^-3^ | 1.4±0.5×10^-3^ | 1.7±0.8×10^-3^ | 5.7±0.8×10^-3^ |
|  | OSKM | 1.3±0.4×10^-3^ | 1.9±0.9×10^-3^ | 3.7±0.7×10^-3^ | 4.6±0.6×10^-3^ | 3.9±0.9×10^-3^ | 6.1±0.5×10^-3^ |
| en-Sox2 | OSKCtr | 1.2±0.3×10^-3^ | 1.1±0.5×10^-3^ | 1.4±0.7×10^-3^ | 2.0±2.3×10^-3^ | 7.4±0.3×10^-3^ | 8.6±0.3×10^-3^ |
|  | OSKR | 2.1±0.8×10^-3^ | 1.7±0.5×10^-3^ | 2.0±0.9×10^-3^ | 1.6±0.3×10^-3^ | 5.0±0.7×10^-3^ | 2.7±0.4×10^-2^ |
|  | OSKM | 2.5±0.4×10^-3^ | 2.7±0.3×10^-3^ | 3.6±0.5×10^-3^ | 6.3±0.7×10^-3^ | 1.8±0.5×10^-2^ | 1.6±0.2×10^-2^ |
| en-Klf4 | OSKCtr | 8.5±0.7×10^-3^(A) | 7.4±1.0×10^-3^(A) | 4.5±2.0×10^-3^(A) | 6.3±2.3×10^-3^(A) | 6.7±1.3×10^-3^(A) | 6.1±0.5×10^-3^(A) |
|  | OSKR | 1.6±0.2×10^-2^(B) | 1.1±0.5×10^-2^(b,B) | 1.4±0.3×10^-2^(ab,B) | 4.3±0.6×10^-2^(c,B) | 2.2±0.5×10^-2^(d,B) | 7.9±1.8×10^-3^(be,B) |
|  | OSKM | 6.8±3.0×10^-3^(A) | 5.2±0.6×10^-3^(A) | 3.9±1.4×10^-3^(A) | 5.3±0.9×10^-3^(A) | 3.7±0.7×10^-3^(A) | 4.5±0.8×10^-3^(A) |
| en-c-Myc | OSKCtr | 4.7±0.7×10^-3^(a,A) | 3.6±0.6×10^-3^(b,A) | 3.9±0.4×10^-3^(b,A) | 3.1±0.6×10^-3^(c,A) | 4.1±0.5×10^-3^(b,A) | 2.5±0.4×10^-3^(d,A) |
|  | OSKR | 5.0±0.4×10^-3^(a,A) | 2.3±0.5×10^-3^(b,B) | 2.2±0.7×10^-3^(b,B) | 5.5±0.6×10^-3^(b,B) | 5.9±0.2×10^-3^(ac,B) | 1.9±0.6×10^-3^(b,A) |
|  | OSKM | 2.2±0.5×10^-3^(a,B) | 3.3±0.2×10^-4^(b,C) | 3.5±0.5×10^-4^(b,C) | 4.8±2.0×10^-4^(a,C) | 6.1±3.6×10^-4^(b,C) | 7.5±4.5×10^-4^(d,B) |
| en-Rab32 | OSKCtr | 1.2±0.3×10^-2^ | 2.3±0.3×10^-2^ | 2.4±0.9×10^-2^ | 2.4±0.4×10^-2^ | 1.9±0.2×10^-2^ | 2.0±0.5×10^-2^ |
|  | OSKR | 9.0±0.6×10^-3^ | 2.3±0.3×10^-2^ | 1.8±0.1×10^-2^ | 2.3±0.5×10^-2^ | 1.5±0.3×10^-2^ | 1.2±0.4×10^-2^ |
|  | OSKM | 4.9±0.5×10^-2^ | 3.5±0.4×10^-2^ | 3.2±0.5×10^-2^ | 2.7±0.7×10^-2^ | 2.0±0.4×10^-2^ | 2.4±0.5×10^-2^ |
| Esrrb | OSKCtr | 1.6±0.2×10^-3^(a) | 2.8±0.8×10^-3^(a) | 1.3±0.6×10^-2^(b) | 3.9±0.8×10^-2^(c,A) | 7.6±0.6×10^-2^(d,A) | 5.5±0.9×10^-2^(c,A) |
|  | OSKR | 1.7±0.7×10^-4^(a) | 1.2±0.9×10^-3^(a) | 2.1±0.4×10^-2^(b) | 5.5±0.6×10^-2^(c,B) | 7.2±0.8×10^-2^(d,A) | 1.7±0.2×10^-1^(e,B) |
|  | OSKM | 1.7±0.4×10^-3^(a) | 5.2±0.6×10^-3^(a) | 2.7±0.6×10^-2^(b) | 7.0±0.9×10^-2^(c,B) | 1.4±0.1×10^-1^(d,B) | 1.2±0.2×10^-1^(e,C) |
| Nr5a2 | OSKCtr | 2.6±0.5×10^-4^(a) | 1.4±0.6×10^-3^(b) | 1.7±0.7×10^-3^(b) | 2.3±0.9×10^-3^(b,A) | 4.0±0.5×10^-3^(c) | 4.7±0.4×10^-3^(d,A) |
|  | OSKR | 2.3±0.4×10^-4^(a) | 9.7±1.8×10^-4^(a) | 7.7±0.9×10^-4^(a) | 3.4±0.6×10^-3^(b,B) | 4.7±0.6×10^-3^(c) | 1.1±0.8×10^-2^(d,B) |
|  | OSKM | 4.2±0.3×10^-4^(a) | 4.8±0.5×10^-4^(a) | 1.6±0.3×10^-3^(b) | 2.1±0.5×10^-3^(b,A) | 5.3±0.6×10^-3^(c) | 7.9±1.0×10^-3^(d,C) |
| Klf2 | OSKCtr | 4.8±0.9×10^-3^(a) | 3.0±0.6×10^-3^(a) | 8.6±0.7×10^-3^(a) | 7.8±0.8×10^-3^(a) | 6.7±0.7×10^-3^(a) | 8.1±1.4×10^-3^(a) |
|  | OSKR | 4.6±0.3×10^-3^(a) | 8.5±2.3×10^-3^(a) | 9.0±1.0×10^-3^(a) | 1.7±0.2×10^-2^(a) | 1.1±0.2×10^-2^(a) | 1.8±0.2×10^-2^(a) |
|  | OSKM | 4.4±0.2×10^-3^(a) | 5.9±1.0×10^-3^(a) | 6.9±0.8×10^-3^(a) | 6.4±0.7×10^-3^(a) | 5.7±0.6×10^-3^(a) | 9.9±1.0×10^-3^(a) |
| Tbx3 | OSKCtr | 9.8±1.0×10^-3^(a) | 1.1±0.5×10^-2^(a) | 9.3±1.0×10^-3^(a) | 1.1±0.5×10^-2^(a,A) | 9.6±0.6×10^-3^(a,A) | 1.3±0.2×10^-2^(a) |
|  | OSKR | 1.1±0.4×10^-2^(a) | 1.4±0.3×10^-2^(a) | 1.4±0.2×10^-2^(a) | 3.2±0.5×10^-2^(b,B) | 2.7±0.4×10^-2^(c,B) | 1.6±0.2×10^-2^(a) |
|  | OSKM | 1.0±0.2×10^-2^(a) | 1.1±0.2×10^-2^(a) | 1.0±1.0×10^-2^(a) | 1.2±0.4×10^-2^(a,A) | 1.4±0.2×10^-2^(a,A) | 1.4±0.3×10^-2^(a) |
| Nanog | OSKCtr | 6.2±1.0×10^-4^ | 4.9±0.4×10^-4^ | 2.3±0.9×10^-3^ | 2.5±0.2×10^-3^ | 3.2±0.5×10^-3^ | 5.5±0.1×10^-3^ |
|  | OSKR | 4.1±0.8×10^-4^ | 4.8±0.8×10^-4^ | 3.7±0.2×10^-4^ | 3.5±0.5×10^-4^ | 1.5±0.2×10^-3^ | 4.6±0.6×10^-3^ |
|  | OSKM | 5.1±0.6×10^-4^ | 3.8±0.9×10^-4^ | 8.4±0.6×10^-4^ | 1.9±0.8×10^-3^ | 4.9±0.5×10^-3^ | 4.0±0.5×10^-3^ |
| Eomes | OSKCtr | 1.9±0.6×10^-4^ | 1.8±0.5×10^-4^ | 3.2±0.8×10^-4^ | 4.9±1.0×10^-4^ | 6.6±0.7×10^-4^ | 8.0±1.3×10^-4^ |
|  | OSKR | 1.6±0.7×10^-4^ | 2.0±0.7×10^-4^ | 2.8±1.0×10^-4^ | 5.2±0.7×10^-4^ | 7.1±0.2×10^-4^ | 5.2±1.0×10^-4^ |
|  | OSKM | 2.7±1.1×10^-4^ | 5.4±0.2×10^-4^ | 7.4±0.9×10^-4^ | 1.1±0.2×10^-3^ | 1.9±0.4×10^-3^ | 1.5±0.3×10^-3^ |
| Sall4 | OSKCtr | 5.7±0.6×10^-5^(a) | 5.7±0.8×10^-5^(a) | 6.9±0.9×10^-5^(a,A) | 1.6±0.6×10^-4^(b,A) | 4.4±0.9×10^-3^(c,A) | 3.4±0.4×10^-3^(d,A) |
|  | OSKR | 1.1±0.9×10^-4^(a) | 7.5±0.9×10^-5^(a) | 6.2±1.0×10^-5^(a,A) | 1.8±0.8×10^-4^(a,A) | 3.3±0.7×10^-3^(b,A) | 8.8±0.8×10^-3^(c,B) |
|  | OSKM | 2.0±0.4×10^-4^(a) | 1.5±0.5×10^-4^(a) | 2.1±0.1×10^-3^(b,B) | 5.3±0.6×10^-3^(c,B) | 1.2±0.3×10^-3^(d,B) | 5.4±0.6×10^-3^(c,C) |

The gene expression profiles were expressed using the comparative CT (2^-ΔCT^) method. The ΔCT was calculated using β-actin, EF1-α, and α-tubulin as internal controls. Data were shown as M ± SD, n=3. The mean values of each group were analyzed by Duncan's multiple range tests. Small letters means significant at 0.05 level compared by the means in the same row, and capital letters means significant at 0.05 level compared by the means in the same column.

**Supplementary Table 5. Primers used in this experiment**

| β-Actin-F | AGATCAAGATCATTGCTCCTCCT | Fis1-F | CAGTGTTGCGTGTTAAGGGATG |
| --- | --- | --- | --- |
| β-Actin-R | ACGCAGCTCAGTAACAGTCC | Fis1-R | TTCAAAATTCCTTGCAGCTTCGT |
| α-Tubulin-F | AAGCAGCAACCATGCGTGAG | Mfn-1-F | ATTGCCACAAGCTGTGTTCG |
| α-Tubulin-R | CCTCCCCCAATGGTCTTGTC | Mfn-1-R | CTAGGGACCTGAAAGATGGGC |
| β-Tubulin-F | TGAGGCCTCCTCTCACAAGTA | Mfn-2-F | GGAGTCAACACCATCAGGGG |
| β-Tubulin-R | CCGCACGACATCTAGGACTG | Mfn-2-R | CAGAGGAGAAGTTTCTAGCTGGT |
| EF1α-F | GTGTTGTGAAAACCACCGCT | MFF-F | TCAAAGCGAAGAGAGCCGAG |
| EF1α-R | AGGAGCCCTTTCCCATCTCA | MFF-R | CCACGGGCAGAGGAAAGATT |
| B2m-F | TGCTATCCAGAAAACCCCTCA | Tom20-F | CGATCGGTCGGAGCTAACC |
| B2m-R | TTTCAATGTGAGGCGGGTGG | Tom20-R | TTTCTTCGTTCTCGAAGCCTGT |
| pMX-1811s | GACGGCATCGCAGCTTGGATACAC | Opa1-F | CTGCAGGTCCCAAATTGGTT |
| Oct4 Rv | CCAATACCTCTGAGCCTGGTCCGAT | Opa1-R | TCTTTGTCTGACACCTTCCTGT |
| Sox2 Rv | GCTTCAGCTCCGTCTCCATCATGTT | Mtor-F | AAGCCGCGCGAACCTC |
| Klf4-943Rv | GTGGGTTAGCGAGTTGGA | Mtor-R | TCTTCAGTCCACTGGCGAAC |
| c-Myc Rv | TCGTCGCAGATGAAATAGGGCTG | Tsc1-F | ATGGCCCAGTTAGCCAACATT |
| ex-Rab32-R | GTATACTCGAGTCATGTTGCCAAAC | Tsc1-R | CAGAATTGAGGGACTCCTTGAAG |
| en-Oct4-F | AGTGGGGCGGTTTTGAGTAA | Tsc2-F | GTTCCCGTGCTAACAGCATTA |
| en-Oct4-R | TTCCAAAGAGAACGCCCAGG | Tsc2-R | TGGCACAGCGGTAAATAAGGC |
| en-Klf4-F | ACCCCTCTCTCTTCTTCGGA | Akt-F | ATGAACGACGTAGCCATTGTG |
| en-Klf4-R | ACGCCAACGGTTAGTCGG | Akt-R | TTGTAGCCAATAAAGGTGCCAT |
| en-Sox2-F | GATCAGCATGTACCTCCCCG | Gsk3β-F | TTCTTGCGGGAGAACTTAATGC |
| en-Sox2-R | TCCTCTTTTTGCACCCCTCC | Gsk3β-R | CTTGCTGCCATCTTTATCTCTGCT |
| en-Rab32-F | ATCTGTCAGGTCGGCCTTTG | Ampkα1-F | GTCAAAGCCGACCCAATGATA |
| en-Rab32-R | GTTGCCAAACCGTTCCTGTC | Ampkα1-R | CGTACACGCAAATAATAGGGGTT |
| Oct4-F | GGCGTTCTCTTTGGAAAGGTG | Lipin1-F | CCTCTTGAACACATTGGAGCC |
| Oct4-R | AGTTCGCTTTCTCTTCCGGG | Lipin1-R | GAGCTCCTTCACCGTCACAA |
| Sox2-F | CCCACCTACAGCATGTCCTAC | Acox2-F | TCCCAGGACAGAGGTGAGTC |
| Sox2-R | AGTGGGAGGAAGAGGTAACCA | Acox2-R | CGGGAGGTACCAAGAACCTTT |
| Klf4-F | GACTAACCGTTGGCGTGAGG | Etfdh-F | CGGCTTGCAGTCCATACCAA |
| Klf4-R | GTCTAGGTCCAGGAGGTCGT | Etfdh-R | TCTGCTGCCAAACTTCCACT |
| cMyc-F | CAGCGACTCTGAAGAAGAGCA | Hmgcs2-F | AGAGGCCTTCAGGGGTCTAA |
| cMyc-R | TTGTGCTGGTGAGTGGAGAC | Hmgcs2-R | GGTAAAGGGAGGCCTTGGTC |
| Ldha-F | AACTTGGCGCTCTACTTGCT | Acacb-F | ACCTGCAGAACGAAGGTGAG |
| Ldha-R | GGACTTTGAATCTTTTGAGACCTTG | Acacb-R | GACTCCTCGATCTTGAGCGG |
| Pgam1-F | ATCTCGGCGATCCTCAGTTG | Fasn-F | GTGATAGCCGGTATGTCGGG |
| Pgam1-R | TTCATAGCCAGCATCTCGCA | Fasn-R | TAGAGCCCAGCCTTCCATCT |
| Cox4i1-F | GTGGCAGAATGTTGGCTTCC | Acaca-F | TGGGCGGAATGGTCTCTTTC |
| Cox4i1-R | ACACTCCCATGTGCTCGAAG | Acaca-R | GTTCATCCCTGGGGACCTTG |
| Atp5B-F | ACCTCGGTGCAGGCTATCTA | Mlycd-F | CCGAGCAGAGCGCCG |
| Atp5B-R | AATAGCCCGGGACAACACAG | Mlycd-R | TGATGTGATGGAAGAGGCCG |
| Drp1-F | GGAGAAGAAAATGGAGTTGAAGCA | Spot14-F | TGAGAACGACGCTGCTGAAA |
| Drp1-R | CGTTGGGCGAGAAAACCTTG | Spot14-R | TGGTATTTCCGCGTCACCTC |
| Mig12 -F | GTGCAAGAGCAGAAAAAGGCT | PI3K-F | CACCATTACAAAGAAAGCCGGA |
| Hk2-F | GTTTCTGGAAACTTGAGGCCC | PI3K-R | TTCTTCCCTTGAGATGTCTCCC |
| Hk2-R | GGTCAACCTTCTGCACTTGG | Erk-F | CCCAAGTGATGAGCCCATTG |
| Uqcr2-F | AGTCAATGGCAGCAAGTGGA | Erk-R | TCAATGGAAGGGGACAAACTGA |
| Uqcr2-R | ATGTGTTTGCTGCTCTGGGC | Lkb1-F | GACTCCGAGACCTTATGCCG |
| Ulk1-F | GTGCACAATTACCAGCGCAT | Lkb1-R | GCGGAAGTACCCATGAGCTT |
| Ulk1-R | ATGGTTCCCACTTGGGGAGA | Redd1-F | GGGATCGTTTCTCGTCCTCC |
| Atg3-F | TCCAACATGGCAATGGGCTA | Redd1-R | ATGAGGAGTCTTCCTCCGGC |
| Atg3-R | TACACCGCTTGTAGCATGGA | Tbc1d1-F | CGTCTCGGGACGATGCC |
| Atg4a-F | GGCCTTGGGATTTTTCTGCAA | Tbc1d1-R | ATGATCTCAGGCACCTTGGC |
| Atg4a-R | AAAGGGAGGCCAGTGTGATG | Plin1-F | CTGTCTGAGACTGAGGTGGC |
| Atg4b-F | GACAGGGAAGATGGACGCAG | Plin1-R | TTCTCCTGCTCAGGGAGGTC |
| Atg4b-R | TAGGGCCAGTTCCCCCAATA | Plin2-F | GTGTGGTGATCTGGACCGTG |
| Atg5-F | TGCGGTTCACTCTGGTTCC | Plin2-R | CTCATCACCACGCTCTGTTG |
| Atg5-R | CCAAAGCCAAACCGAGGTG | Plin3-F | AAAGGCGTCAAGACCCTCAC |
| Atg7-F | GTTGAGCGGCGACAGCATT | Plin3-R | CGCCAAAACCTTCTCAGTCG |
| Atg7-R | TGTCAGGTGCTCACATCCAG | Plin4-F | TGAGGGACCCTTTACCCTGA |
| Map1lc3a-F | ATCATCGAGCGCTACAAGGG | Plin4-R | AGGCAGGGACCCAAAGAAAC |
| Map1lc3a-R | AGCCGAAGGTTTCTTGGGAG | Plin5-F | ACTTTCCTGCCCGTCAAAGG |
| Map1lc3b-F | AAGAGTGGAAGATGTCCGGC | Plin5-R | TCTGATCACCGGACATTCTGC |
| Map1lc3b-R | AAGAGTGGAAGATGTCCGGC |  |  |
| Ulk1-F | GCATCGAGCAAAACCTGCAA |  |  |
| Ulk1-R | GTTCCCACTTGGGGAGAAGG |  |  |

**Supplementary Methods**

**Derivation of Mouse Embryonic** **Fibroblast Cells (MEFs)**

MEFs were isolated from 13.5 day embryos heterozygous for the Oct4-GFP/ RFP mitochondria / EGFP transgenic allele. Gonads and internal organs were removed when the embryos were processed for isolation of MEFs. Isolated MEFs in early passages (up to passage 4) were used for further experiments. Secondary embryonic ﬁbroblasts that carry the OSKM reprogramming factors (TF4) were a gift from Dr. Weiming Ruan.

**Gene-screening**

MEFs at passage 3 were seeded onto on feeder cells in six-well plates at a density of 8,000 per well in MEF medium (Dulbecco’s modiﬁed Eagle’s medium (DMEM) supplemented with 10% FBS, L-glutamine and nonessential amino acids). Cells were transduced with pMXs retroviruses[^1^](#_ENREF_1) carrying the Oct4, Sox2, Klf4 and candidate genes. Two days later, the medium was changed into mES medium (DMEM supplemented with 15% FBS, L-glutamine, nonessential amino acids, β-mercaptoethanol, and 1,000 U/ml LIF). The medium used from day 6 to 18 was mES medium or 10% KOSR (DMEM supplemented with 10% KnockOut SR XenoFree CTS, L-glutamine, nonessential amino acids, β-mercaptoethanol, and 1,000 U/ml LIF. The medium was changed based on the reported article[^2^](#_ENREF_2)). The medium was changed every other day. For quantiﬁcation of reprogramming efﬁciency using the Oct4-GFP reporter, the transfected MEFs cultures on day 16 were scanned under a ﬂuorescence microscope in Biostation (Nikon) and scored for GFP^+^ colonies. On day 18 cells were ﬁxed and stained for alkaline phosphatase activity using an alkaline phosphatase detection kit (Sigma, catalog No. 85L3R). The plates were then photographed and the numbers of AP colonies were counted independently according to the photos.

**Identification of cell proliferation**

MEFs were transfected with control vector (MEFCtr) or Rab32 (MEFOER), and then the cells were cultured in Biostation CT (Nikon) for 5 days following transfection. Cell images were automatically acquired every 12 hours by a BioStation CT and the system’s software calculated the cell number to identify the proliferation of MEFs,

**FACS Analysis**

OG2 MEFs which were retroviral transduced with OSKCt, OSKR, OSKM for 7 days, were trypsinized, washed once in PBS, and resuspended in 250 μL 10% KOSR medium. Cell fluorescence was analyzed using a Beckman MoFlo XDP cell sorter.

**Construction of Vectors**

For overexpression of the candidate protein, the coding sequence of full-length mouse candidate genes was cloned into the pMXs retroviral vector. The short hairpin RNA (shRNA) targeting mouse Rab32 mRNA or the control shRNA was cloned into the plvth lentivirus retroviral vector. The sequence of shRab32 was GCATGACTCGAGTATACTA. The sequence of luciferase was GATGAAATGGGTAAGTCA.

**Retrovirus Production and Generation of iPSCs**

Retrovirus were produced by transfection of plat-E cells with pMXs retroviral vectors containing coding sequences of mouse Oct4, Sox2, Klf4 and candidate genes, using X-tremeGENE 9 DNA transfection reagent (Roche). Medium of the plat-E cells transfected with the exogenousgene was filtrated through 0.45µm filters, These filtrated medium were then added to the six-well plates seeded at a density of 8,000 cells per well on the feeder layer. Polybrene was used at a ﬁnal concentration of 8 µg/ml to facilitate the transduction. Medium was changed after 12 h virus transduction and this day is termed ‘‘day 0.’’

To generate fully reprogrammed iPSCs, ES-like colonies were picked on day 16, digested into single cell suspensions, and plated in 48-well plates with mES medium. Oct4-GFP positive colonies with homogenous morphologies were further characterized.

**Embryoid Body Formation of iPSCs**

The iPSCs were grown to approximately 80% conﬂuence and dissociated into single cells in the differentiation medium (DMEM supplemented with 15% FBS, L-glutamine, nonessential amino acids, and β-mercaptoethanol). They were seeded at a density of 1×10^5^ cells per uncoated 3.5 cm Petri dish. The dishes were taped to a rotary platform in the incubator. Medium was changed every 2 days. Embryoid bodies were collected on day 3 for further differentiation on the uncoated Petri dishes in the differentiation medium.

**RNA Purification and cDNA Preparation**

Total RNA was extracted from cells using Trizol reagent according to the manufacturer’s instructions (Invitrogen). The RNA was reverse-transcribed using oligo-dT and M-MLV Reverse Transcriptase (Promega, Madison, WI).

**Real-Time PCR**

Quantitative Real-Time PCR was run on a Light Cycler 480 II Real-Time PCR System (Roche, Basel, Switzerland) using the Light Cycler 480 SYBR Green I Master (Roche, Basel, Switzerland). Primer sequences for this section are listed in Supporting Information Table S3.

**Teratoma Production and Analysis**

Approximately 1×10^6^ iPSCs were suspended in 150 µl PBS buffer and injected into NOD-SCID mice to form teratomas. Three weeks after injection, the teratomas were harvested, ﬁxed overnight with 4% paraformaldehyde, embedded in parafﬁn, sectioned, HE stained, and analyzed.

**Production of Chimeric Mice**

iPSCs (with a C57/BL6 background) were injected into 8-cell embryos which were isolated from superovulated female CD-1 mice. The reconstructed embryos were cultured *in vitro* and developed into blastocysts. Chimeras were produced by transplantation of injected blastocysts into uterus of pseudopregnant CD-1 mice.

**Immunofuorescence**

Cells were fixed with 4% paraformaldehyde, permeabilized with 0.1% Triton X-100, and blocked with 2% BSA (Sigma). The cells were then stained with primary antibodies against Oct4 (abcam), Sox2 (abcam), Nanog (abcam), SSEA1 (abcam), Nestin (abcam), Gata6 (abcam), α- Smooth Muscle Actin (abcam), Rab32 (biorbyt), and LC3 (MBL), followed by staining with the respective secondary antibodies conjugated to Alexa Fluor (Invitrogen). The cells were counterstained with DAPI (Sigma).

**Generation of iPSCs, Immunofluorescent Staining and** **High-Throughput Imaging**

EGFP-MEFs at passage 3 were seeded onto 96-well View Plates (PerkinElmer, Waltham, MA) at a density of 800 cells per well on feeder cells in MEF medium. Cells were transfected by the medium containing retroviruses for: a) Oct4, Sox2, Klf4 and control vector (OSKCtr); b) Oct4, Sox2, Klf4 and Rab32 (OSKR); and c) Oct4, Sox2, Klf4 and c-Myc (OSKM). 10% KOSR medium was used from day 6 to the end.

**Lyso-Tracker**

Removed the medium from the dish and add the prewarmed (37°C) LysoTracker-containing medium (50nM, Invitrogen, L12492). Incubate the cells for 50 min under growth conditions. Then cells were stained with 0.2 µg/mL Hoechst33342 for 5 min in incubator, washed three times with PBS medium, and next cultured in fresh medium.

**Nile red staining**

Cells were fixed with 4% paraformaldehyde, washed three times with PBS medium, then stained the cell with PBS medium containing the Nile red (25 µg/mL, Invitrogen, N-1142) in dark for 5 min, and 0.2 µg/mL Hoechst 33342 stain for 5 min in dark, followed by washing three times with PBS medium.

**Generation of G4OERC cells**

For overexpression of Rab32, coding sequence of full length mouse candidate genes was cloned into the inducible PiggyBac vector (PBQM812A-1, SBI). Electrotransfection was performed using Nucleofector Kits for Mouse Embryonic Stem Cells (Lonza) following manufacturer’s protocol. To generate mutant G4 cells, 1×10^6^ cells were transfected with 4 ug overexpression plasmid using A-030 program, and 24 h later, we used 1 μg/mL puromycin to eliminate the negative cells.

**Supplementary References**

1. Takahashi, K. & Yamanaka, S. Induction of pluripotent stem cells from mouse embryonic and adult fibroblast cultures by defined factors. *Cell* **126**, 663-676 (2006).

2. Chen, J. *et al.* H3K9 methylation is a barrier during somatic cell reprogramming into iPSCs. *Nat Genet* **45**, 34-42 (2013).
